# Supplementary material for: Current provision of myelopathy education in medical schools in the UK: protocol for a national medical student survey
Source: BMJ Open. 2020 Aug 26;10(8):e035563. doi: 10.1136/bmjopen-2019-035563 (PMC7451530; doi:10.1136/bmjopen-2019-035563)
Supplement: Author's manuscript [file bmjopen-2019-035563.draft_revisions.pdf]

BMJ Open is committed to open peer review. As part of this commitment we make the peer review history of every article we publish publicly available.

When an article is published we post the peer reviewers' comments and the authors' responses online. We also post the versions of the paper that were used during peer review. These are the versions that the peer review comments apply to.

The versions of the paper that follow are the versions that were submitted during the peer review process. They are not the versions of record or the final published versions. They should not be cited or distributed as the published version of this manuscript.

BMJ Open is an open access journal and the full, final, typeset and author-corrected version of record of the manuscript is available on our site with no access controls, subscription charges or pay-per-view fees (http://bmjopen.bmj.com).

If you have any questions on BMJ Open's open peer review process please email info.bmjopen@bmj.com

BMJ Open

Current Provision of Myelopathy Education in Medical Schools in the United Kingdom: Protocol for a National Medical Student Survey

Journal:	*BMJ Open*
Manuscript ID	bmjopen-2019-035563
Article Type:	Protocol
Date Submitted by the Author:	08-Nov-2019
Complete List of Authors:	Mowforth, Oliver; University of Cambridge, Division of Neurosurgery, Department of Clinical Neurosciences<br>Davies, Benjamin; University of Cambridge, Division of Neurosurgery, Department of Clinical Neurosciences<br>Stewart, Max; University of Cambridge, School of Clinical Medicine<br>Smith, Sam; University of Cambridge, School of Clinical Medicine<br>Willison, Alice; University of Dundee, Ninewells Hospital and Medical School<br>Ahmed, Shahzaib; University of Cambridge, School of Clinical Medicine<br>Sadler, Iwan; Myelopathy.org<br>Sarewitz, Ellen; Myelopathy.org<br>Stacpoole, Sybil; University of Cambridge, Neurology Unit, Department of Clinical Neurosciences<br>Kotter, Mark; University of Cambridge, Department of Clinical Neurosurgery; Ann McLaren Laboratory of Regenerative Medicine, Department of Clinical Neurosciences
Keywords:	NEUROSURGERY, Neurosurgery < SURGERY, MEDICAL EDUCATION & TRAINING, NEUROLOGY

SCHOLARONE™
Manuscripts

I, the Submitting Author has the right to grant and does grant on behalf of all authors of the Work (as defined in the below author licence), an exclusive licence and/or a non-exclusive licence for contributions from authors who are: i) UK Crown employees; ii) where BMJ has agreed a CC-BY licence shall apply, and/or iii) in accordance with the terms applicable for US Federal Government officers or employees acting as part of their official duties; on a worldwide, perpetual, irrevocable, royalty-free basis to BMJ Publishing Group Ltd ("BMJ") its licensees and where the relevant Journal is co-owned by BMJ to the co-owners of the Journal, to publish the Work in this journal and any other BMJ products and to exploit all rights, as set out in our licence.

The Submitting Author accepts and understands that any supply made under these terms is made by BMJ to the Submitting Author unless you are acting as an employee on behalf of your employer or a postgraduate student of an affiliated institution which is paying any applicable article publishing charge ("APC") for Open Access articles. Where the Submitting Author wishes to make the Work available on an Open Access basis (and intends to pay the relevant APC), the terms of reuse of such Open Access shall be governed by a Creative Commons licence – details of these licences and which Creative Commons licence will apply to this Work are set out in our licence referred to above.

Other than as permitted in any relevant BMJ Author's Self Archiving Policies, I confirm this Work has not been accepted for publication elsewhere, is not being considered for publication elsewhere and does not duplicate material already published. I confirm all authors consent to publication of this Work and authorise the granting of this licence.

For peer review only

Current Provision of Myelopathy Education in Medical Schools in the United Kingdom: Protocol for a National Medical Student Survey

Oliver D. Mowforth B.A. M.B. B.Chir,[1] Benjamin M. Davies B.Sc. MRCS,[1] Max Stewart B.A,[2] Sam Smith B.A.,[2] Alice Willison B.Sc. MRes MBChB,[3], Shahzaib Ahmed B.A.,[2] Iwan Sadler,[4] Ellen Sarewitz M.A.,[4] Sybil R. Stacpoole Ph.D.,[5], Mark R. Kotter M.D. M.Phil Ph.D.[1,6]

1. Division of Neurosurgery, Department of Clinical Neurosciences, University of Cambridge, Cambridge, UK
2. School of Clinical Medicine, University of Cambridge, Cambridge, UK
3. Ninewells Hospital and Medical School, University of Dundee, Dundee, UK
4. Myelopathy.org, Cambridge, UK
5. Neurology Unit, Department of Clinical Neurosciences, University of Cambridge, Cambridge, UK
6. Anne McLaren Laboratory for Regenerative Medicine, Wellcome Trust-Medical Research Council Cambridge Stem Cell Institute, University of Cambridge, Cambridge, UK.

Key words: spinal cord diseases; cervical vertebrae; spondylosis; spinal osteophytosis; surveys and questionnaires; students, medical; education, medical

Corresponding author:

Dr Oliver Mowforth
Division of Neurosurgery
Department of Clinical Neurosciences
University of Cambridge
CB2 0SZ
Email: om283@cam.ac.uk
Telephone: +44 1223 763366
Fax: +44 1223 763350

Reference count: 35

Full text word count: 2550

Figure count: 5

For peer review only

Abstract

Introduction

Degenerative cervical myelopathy (DCM) is a common, disabling and progressive neurological condition triggered by chronic compression of the cervical spinal cord by surrounding degenerative changes. Early diagnosis and specialist management are essential to reduce disability, yet time to diagnosis is typically prolonged. Lack of sufficient representation of DCM in undergraduate and post-graduate medical curricula may contribute to the poor recognition of DCM by non-specialist doctors in clinical practice.

In this study our objective therefore is to assess DCM teaching provision in medical schools throughout the United Kingdom (UK) and to assess the impact of teaching on the DCM knowledge of UK medical students.

Methods

A 19-item questionnaire capturing data on medical student demographics, myelopathy teaching and myelopathy knowledge was designed. Ethical approval was granted by the Psychology Research Ethics Committee, University of Cambridge. An online survey was hosted on Myelopathy.org, an international myelopathy charity. Students studying at a UK medical school are eligible for inclusion. The survey is advertised nationally through university social media pages, university email bulletins and the national student networks of the Neurology and Neurosurgery Interest Group (NANSIG) and Myelopathy.org. Advertisements are scheduled monthly over a 12-month recruitment period. Participation is incentivised by entering consenting participants of completed surveys to an Amazon voucher prize draw. Responses are anonymised using participant-chosen unique identifier codes. A participant information sheet followed by an explicit survey question captured participant informed consent. The study is due to report in Spring 2020. Regular updates on the progress of the study will be published on Myelopathy.org.

Conclusion

This study will provide national data on UK myelopathy education and any relationships that exist between teaching provision and medical student knowledge of DCM. The study may identify training and/or knowledge deficiencies that could help inform the design of future curricula.

For peer review only

Strengths and limitations of this study

- This is a large national questionnaire-based study of all medical student in the United Kingdom
- This is the first study to assess DCM medical education in the UK
- This is a student-led study with oversight from senior researchers and involvement of DCM patients
- The study data will provide insight into an area that has potential to translate into substantial improvements in patient outcomes
- Risk of selection bias towards may overestimate the current knowledge of DCM among UK medical students

For peer review only

Introduction

Degenerative cervical myelopathy (DCM) is a common neurological condition of symptomatic cervical spinal cord compression due to degenerative changes of the surrounding spine (Figure 1).[1] DCM is the most common cause of spinal cord impairment in adults worldwide with an estimated prevalence of up to 5% in the over 40s.[1],[2],[3].

Substantial improvements in the care of DCM patients could be realised today without any additional scientific understanding of the condition.[1] DCM patients almost universally experience diagnostic delays. For example, one study demonstrated that on average it took 2 years and 5 clinic appointments before the diagnosis was made.[4] Improvement in diagnosis is therefore a key target which would provide immediate reductions in disability.[5]

Initially, DCM patients are commonly seen in primary care. Other triage points include many other medical services, including emergency and medical physicians.[6],[7] These non-specialist triage points appear key to earlier diagnosis, yet DCM features poorly in medical curricula.[8] This risks non-specialist doctors being poorly equipped to detect DCM.

The current focus and makeup of DCM medical education is unclear. Our recent evaluation of undergraduate and post-graduate medical curricula identified deficiencies in DCM representation, however collectively this did not correlate with performance in multiple-choice question banks.[8]

The aim of this study is to describe the teaching of DCM in UK medical schools and to assess the influence of this on medical student knowledge of DCM. Ultimately, the aim is to identify both training and knowledge gaps to inform guidance on DCM education in order to address the burden and impact of delayed diagnosis. We hypothesise that knowledge and awareness amongst medical professionals is related to their specific medical education on DCM.

For peer review only

Methods

Study Partners

Myelopathy.org

Myelopathy.org is an international cervical myelopathy charity, which aims to improve health and well-being in DCM.[9] This study forms part of a wider project to evaluate DCM education and awareness amongst medical professionals in the UK, and develop focused and effective interventions to improve this. This includes our recent gap-analysis of key medical resources and knowledge at each stage of the primary care training pathway[8] and an educational article on DCM for non-specialist medical professionals, commissioned by the British Medical Journal (Figure 2).[1] The charity is focussed on understanding how medical education can be improved as one possible strategy to address the current problems of delayed, missed and under-diagnosis.[1]

Student Society of Myelopathy.org

The foundations of professional knowledge begin with students' formative experiences at medical school. Therefore, the Student Society of Myelopathy.org was established in 2018 with the aim of improving DCM engagement and awareness amongst medical students. The society remains in its infancy but has hosted a series of educational talks at the University of Cambridge; established national medical student essay and research prizes; designed and initiated this current study; and secured sponsorship from corporate partners to support these projects.

Study Design

An electronic survey was agreed to be the most efficient way to simultaneously assess teaching, perceptions of teaching, and current knowledge, and to efficiently reach students from all UK medical schools. Part One of the survey was designed to capture data on basic demographic characteristics and teaching of DCM in UK medical schools. Part Two was designed to capture data on knowledge and awareness of fundamental DCM facts including prevalence, symptoms, diagnostic imaging modalities and surgical effectiveness in addition to appreciation of important current challenges such as prolonged time to diagnosis and poor patient quality of life. Part Two was designed to capture participant perception of their DCM knowledge. These questions were considered to be a reasonable balance of capturing

sufficient data whilst maintaining an acceptably short survey completion time of approximately 5 minutes.

The study management team comprised representation from second year to final year students from multiple UK medical schools. The team comprised an academic neurosurgical registrar [BMD] and an academic neurosurgical consultant [MRK], both with special clinical and academic interests in DCM, and a consultant neurologist [SRS] who is a neuroscience teaching fellow and neuroscience course organiser at the University of Cambridge. Importantly, this is a student-motivated, student-led project with supervision, oversight and mentorship at all stages of the study from a team of senior researchers.

The survey was iteratively refined amongst the study management group until section one was felt to capture sufficient information on current DCM teaching provision in UK medical schools. Similarly, section two was refined until it was felt to evaluate fundamental DCM facts at a level sufficient for a non-specialist, but qualified doctor to appropriately suspect, investigate, diagnose and re-direct care in DCM. In total, a 19-item questionnaire was developed (Figure 3).

Survey Piloting

The survey was piloted in January 2019 by a group of 20 medical students at the University of Cambridge. These students were not directly involved in the design of the study. All students reported the survey to be acceptable on both desktop and mobile devices. No further alterations were therefore made.

Survey Administration

To maximise reach across the UK, the survey was hosted on Qualtrics Survey Software (Provo, United States), a professional online survey platform. Qualtrics Survey Software includes both desktop and mobile-compatible versions, maximising convenience for participants.

The survey comprises two pages. The first page acts as a front page consisting of a participant information sheet and a question capturing informed consent. The second page is the main survey page. It comprises 19 questions over a single page to minimise the numbers of clicks required for participants to complete the survey.

Survey Dissemination

For peer review only

A network of Myelopathy.org Student Representatives was recruited, with representation of students from 25 different medical schools across the UK. Many but not all were also representatives for the Society of British Neurological Surgeons (SBNS) Neurology and Neurosurgery Student Interest Group (NANSIG). A standard advertisement devised by the protocol development team and approved by the research ethics committee is utilised for all advertising (Figure 4). Representatives are sent monthly reminder emails to disseminate adverts at their institution over a 12-month period. The frequency of advertising at each institution is left to the discretion of representatives, guided by local rules.

On completion of the survey participants are invited to leave an email address in a separate Google Forms survey to be entered into a prize draw to win one of five £10 Amazon vouchers. The prize draw was agreed by the study management group and approved by the research ethics committee. The rationale was that prizes of this value may encourage participation without providing an inappropriate incentive to participate.

The survey is advertised in social media groups and on social media pages for relevant student groups and societies at universities across the UK. For example, Facebook groups of surgical, neurosurgical, neurological and other medical student societies were approached. In addition, university mailing lists and email bulletins are being utilised, where appropriate, in accordance with local rules. Adverts are also placed in the monthly NANSIG newsletter, emailed out to medical student subscribers across the UK. The survey link is also hosted on the Student Society of Myelopathy.org webpage and Facebook page.

Eligibility and Representation

All UK medical students are eligible to complete the survey. We aim to secure representation of all UK medical schools.

Consent and Confidentiality

A patient information sheet (PIS) including a question capturing informed consent forms the survey frontpage (Figure 5). The PIS emphasises the rational, purpose, study aims and the voluntariness of participation. By necessity the background information on DCM in the PIS is minimal to avoid compromising the DCM knowledge-based questions in the survey. A page block is utilised to separate the front and main survey pages.

For peer review only

Participants assign themselves a unique identifier based on a defined combination of characters from their mother's maiden name, the street they grew up on and their mobile phone number; this ensures anonymity and confidentiality whilst allowing the possibility of linking together future surveys from the same participant.

Ethical Approval

Ethical Approval was granted by the Psychology Research Ethics Committee, University of Cambridge (PRE.2018.099). Insurance cover was arranged via the University of Cambridge (HVS/2018/2366).

Data Security

All data are stored exclusively on the secure, online Qualtrics platform until closure of the survey. Thereafter, survey data will be extracted directly from the Qualtrics platform into a password-encrypted Excel spreadsheet (Microsoft, California) on a password-protected computer. Only the immediate research team will be granted access to this on a need-to-access basis. After completion of data analysis, all data that is no longer required will be deleted. No participant-identifiable data will be collected or stored at any point.

Statistical Analysis

Data will be analysed in Microsoft Excel and JASP statistical software.

Relationships between duration of DCM teaching, duration of private study on DCM and student perception of their DCM knowledge and objective performance in the DCM knowledge-based questions will be assessed. Differences in performance on the knowledge-based questions will be compared between groups based on medical school, year of medical school, duration of teaching, format of teaching, duration of private study, prior self-reported awareness of DCM, self-rating of DCM knowledge and between groups of medical students wishing to pursue neurological/neurosurgical career specialties and those who do not. Appropriate statistical methods will be selected based on the class and distribution of the data. It is desirable that evaluations will include multi-variate techniques, in order to control for multiple co-variates.

Patient and Public Involvement

Patients were involved in the conception, design, development and conduct of this study. A DCM Patient and Public Engagement day hosted at the University of Cambridge was

For peer review only

captured by Cambridge TV in a documentary.[10] During this day, a focus group of DCM patients emphasised their experiences of diagnostic delays. This has been frequently echoed by patients in Myelopathy.org social media groups and in a DCM word-cloud creating initiative to understand patient's perceptions of the condition for a related project. DCM patients therefore inspired the conception of this study and approved the final survey design. Patients also assisted in the essential administrative tasks of the project, such as logo design. The online survey for the study was hosted on Myelopathy.org, which is a patient-maintained website for people with DCM. Patients were, therefore, active in aiding the disseminating of survey to UK medical students. Patients who were involved in preparation of the manuscript are among the authors. DCM patients are involved in plans to disseminate this research to the patient community, including blog articles on Myelopathy.org, posts in online patient support groups and presence at spinal conferences in the UK.

For peer review only

Discussion

Study Rationale

This is the first study to describe the DCM education at medical schools nationally. Medical students are recruited to an online survey, including an assessment of their DCM knowledge and their personal DCM learning experience. By comparison of learning experience and

knowledge, and also comparison between medical schools, it is anticipated both the presence or absence of a knowledge gap can be evaluated, and the more successful teaching formats identified. This will be used to inform Myelopathy.org education recommendations.

Why assess DCM education?

DCM is a devastating condition with one of the poorest quality of life scores of any chronic disease.[11] Symptoms include loss of manual dexterity, gait impairment, limb paraesthesia, limb weakness, limb stiffness, bladder and bowel disturbance, neck pain and neck stiffness.[1],[12],[13] The full spectrum of symptoms and the natural history of DCM is poorly appreciated,[14], [15] yet it remains clear that the condition poses a substantial burden on the physical and mental health of people with DCM [16] and their supporters [17]. There is a broad differential for DCM symptoms[18] and poor consensus on what the most common DCM symptoms are. In addition, an increasing number of atypical symptoms, such as dysaesthetic sensory symptoms,[6] chest tightness,[19] and headache[20] are being reported.[21] Neurological examination findings include upper motor neuron signs in the upper and lower limbs: spasticity and hyperreflexia in addition to weakness.[1],[13].

The latest international guidelines[22] recommend surgical decompression as the only evidence-based management [23],[24] for patients with moderate to severe, or progressive DCM. Surgery is also a management option in patients with mild DCM.[22] Importantly, surgery can halt progression of the condition but is ineffective at reversing existing damage.[24] Therefore, the traditional medical dogma that prevention is better than cure holds as strongly as ever in DCM: time is spine.[1]

Scientific advances are sorely needed to improve therapeutic options and ultimately to cure disabled patients with DCM.[25], [26] However, whilst research into the neurobiology of DCM and neural regeneration,[27] such as the RECEDE-MYELOPATHY and CSM-PROTECT trials offer hope for the future, [9],[28],[29] even the most optimistic scientist would concede that tangible benefits for patients arising from these technologies remain on the horizon: one systematic review reported average duration of knowledge translation of 17 years from research to practice.[30]

Therefore, pragmatic approaches are required to improving outcomes for today's DCM patients. One such approach may entail addressing the substantial problem of diagnostic delays[1], which is expected to improve outcomes in this progressive condition.[5] One method of instituting this may be through better education. Indeed, a phenomenon of

For peer review only

"neurophobia" is well-described amongst medical students and non-specialist doctors.[31],[32],[33] Additionally, evidence from our recent gap analysis suggests poor representation of DCM in medical curricula.[8] These factors may point to a knowledge gap in DCM medical education.

Benefits of a medical student survey

Assessing teaching and knowledge among medical students is a pragmatic and initial step to evaluate a knowledge gap. Medical school is the foundation of all medical professionals' education. It is likely that this is particularly pertinent for knowledge that this not later core (i.e. built on or refined) during specialisation. For this purpose, a survey of medical students is best placed to simultaneously assess knowledge, whilst reporting on the current provision of DCM teaching and perceptions of it. Moreover, the survey itself may help to prime an audience to support a subsequent intervention, or at the very least raise awareness.

Limitations

Online surveys are an effective method of reaching students from diverse medical schools.[34] In addition, digital collection of data offers a secure and seamless stream, facilitating data analysis. However, their limitation is in understanding how representative the findings are.

In order to mitigate this, a number of different dissemination approaches are being employed. This includes the appointment of local representatives at each UK medical school, the use social media posts, society mailing lists; society newsletters; medical school email bulletins; medical school newsletters, and also the use of prizes as an incentive.[35] Additionally, the capture of participant demographics will help to measure the sample characteristics.

The engagement with open surveys, often involves some predefined interest. It is possible therefore a selection-bias towards students interested in neuroscience is more likely and results would be considered as a best-case scenario. Whilst there is a risk of overlooking or underestimating the magnitude of a knowledge gap given the outlined benefits of this methodology this is an accepted risk. Whatever the relationship between DCM teaching and knowledge turns out to be, it will be instructive with regards to the overall objective of identifying ways of improving DCM diagnosis.

Conclusion

For peer review only

Urgent improvements in outcomes for DCM patients are needed. Earlier diagnosis would improve outcomes based on current treatments. This study aims to evaluate DCM education and knowledge, and explore their relationship, amongst medical students. It is hoped this will inform educational interventions that may offer the greatest chance of shaping the non-specialist doctor's diagnosis DCM of tomorrow.

For peer review only

Author contributions:

ODM, BMD, IS, ES, SRS and MRK were involved in the conceptualisation and design of this study. ODM wrote the first draft of the manuscript. ODM, BMD, MS, SS, AW, SA, IS, ES, SRS and MRK reviewed and approved the final manuscript.

Funding:

Research in the MRNK's laboratory is supported by a core support grant from the Wellcome Trust and MRC to the Wellcome Trust-Medical Research Council Cambridge Stem Cell Institute. MRNK is supported by a NIHR Clinician Scientist Award.

Disclaimer:

This report is independent research arising from a Clinician Scientist Award, CS-2015-15-023, supported by the National Institute for Health Research. The views expressed in this publication are those of the authors and not necessarily those of the NHS, the National Institute for Health Research or the Department of Health and Social Care.

Presented at:

World Federation of Neurosurgical Societies International Meeting, Belgrade, Serbia, March 2019.

British Neurosurgical Research Group Meeting, Edinburgh, November 2019

Competing Interests:

ODM, BMD, MS, SS, AW, SA and MRK have voluntary roles at Myelopathy.org, an international DCM charity.

Patient consent for publication: not required

Ethical approval: approved by Psychology Research Ethics Committee, University of Cambridge

Data availability statement: All data relevant to the study are included in the article or uploaded as online supplementary information.

Acknowledgements:

The authors are grateful to all the patients who have inspired this study, most especially at the University of Cambridge Patient and Public Engagement Day. In addition, the authors would like to thank the Student Society of Myelopathy.org and NANSIG medical student representatives for their assistance in disseminating the research survey.

For peer review only

For peer review only

Figure Legends:

Figure 1. Typical MRI scan from patient with DCM showing spinal cord compression and multi-level degenerative changes.

Figure 2. DCM Educational Article commissioned by the British Medical Journal.

Figure 2. Final Survey Design

Figure 4. A standard advertisement devised by the protocol development team and approved by the research ethics committee is being used for all survey advertising.

Figure 5. Survey Front Page including Patient Information Sheet and Capturing of Informed Consent.

For peer review only

References

1. Davies BM, Mowforth OD, Smith EK, Kotter MR. Degenerative cervical myelopathy. BMJ 2018 Feb 22;360:k186. PMID:29472200

2. Kovalova I, Kerkovsky M, Kadanka Z, Kadanka Z, Nemec M, Jurova B, Dusek L, Jarkovsky J, Bednarik J. Prevalence and Imaging Characteristics of Nonmyelopathic and Myelopathic Spondylotic Cervical Cord Compression: SPINE 2016 Dec;41(24):1908–1916. [doi: 10.1097/BRS.0000000000001842]

3. Nouri A, Tetreault L, Singh A, Karadimas SK, Fehlings MG. Degenerative Cervical Myelopathy: Epidemiology, Genetics, and Pathogenesis. Spine 2015 Jun 15;40(12):E675-693. PMID:25839387

4. Behrbalk E, Salame K, Regev GJ, Keynan O, Boszczyk B, Lidar Z. Delayed diagnosis of cervical spondylotic myelopathy by primary care physicians. Neurosurg Focus 2013 Jul;35(1):E1. PMID:23815245

5. Cates JR, Soriano MM. Cervical spondylotic myelopathy. J Manipulative Physiol Ther 1995 Sep;18(7):471–475. PMID:8568430

6. Mowforth OD, Davies BM, Kotter MR. "I am not delusional!" Sensory dysaesthesia secondary to degenerative cervical myelopathy. BMJ Case Rep 2019 Apr 11;12(4). PMID:30975783

7. Radcliff KE, Curry EP, Trimba R, Walker JB, Purtill JJ, Austin MS, Parvizi J, Vaccaro AR, Hilibrand AS, Albert TJ. High Incidence of Undiagnosed Cervical Myelopathy in Patients With Hip Fracture Compared With Controls. J Orthop Trauma 2016 Apr;30(4):189–193. PMID:26562581

8. Davies B, Wilcock J, Garner J, Waqar M, Kotter M. A quantitative analysis of medical students' and physicians' knowledge of degenerative cervical myelopathy. 2019 Jun 10 [cited 2019 Sep 15]; [doi: 10.17863/CAM.40662]

9. Neurology TL. A focus on patient outcomes in cervical myelopathy. Lancet Neurol 2019 Jul 1;18(7):615. PMID:31202463

10. Roberts E. Elemental Ideas: Cervical Spondylotic Myelopathy. http://www.myelopathy.org/the-csm-documentary.html.

11. Oh T, Lafage R, Lafage V, Protopsaltis T, Challier V, Shaffrey C, Kim HJ, Arnold P, Chapman J, Schwab F, Massicotte E, Yoon T, Bess S, Fehlings M, Smith J, Ames C. Comparing Quality of Life in Cervical Spondylotic Myelopathy with Other Chronic Debilitating Diseases Using the SF-36 Survey. World Neurosurg 2017 Jan 5; PMID:28065875

12. Arnold JG. The clinical manifestations of spondylochondrosis (spondylosis) of the cervical spine. Ann Surg 1955 Jun;141(6):872–889. PMID:14377330

13. Brain WR, Northfield D, Wilkinson M. The neurological manifestations of cervical spondylosis. Brain J Neurol 1952 Jun;75(2):187–225. PMID:14934989

14. Badhiwala JH, Wilson JR. The Natural History of Degenerative Cervical Myelopathy. Neurosurg Clin N Am 2018 Jan;29(1):21–32. PMID:29173433

For peer review only

15. Clair SS, Bell GR. Natural History of Cervical Spondylotic Myelopathy. Semin Spine Surg 2007 Mar 1;19(1):2–5. [doi: 10.1053/j.semss.2007.01.005]

16. Stoffman MR, Roberts MS, King JT. Cervical spondylotic myelopathy, depression, and anxiety: a cohort analysis of 89 patients. Neurosurgery 2005 Aug;57(2):307–313; discussion 307-313. PMID:16094160

17. Mowforth OD, Davies B, Kotter M. Poor quality of life in degenerative cervical myelopathy carers (Preprint). 2019 Jul 25 [cited 2019 Oct 21]; [doi: 10.17863/CAM.42010]

18. Tracy JA, Bartleson JD. Cervical spondylotic myelopathy. The Neurologist 2010 May;16(3):176–187. PMID:20445427

19. Kobayashi H, Kikuchi S, Otani K, Sekiguchi M, Sekiguchi Y, Konno S. Development of a self-administered questionnaire to screen patients for cervical myelopathy. BMC Musculoskelet Disord 2010 Nov 22;11:268. PMID:21092213

20. Schrot RJ, Mathew JS, Li Y, Beckett L, Bae HW, Kim KD. Headache relief after anterior cervical discectomy: post hoc analysis of a randomized investigational device exemption trial. J Neurosurg Spine 2014 Aug;21(2):217–222. PMID:24836655

21. Davies BM, Munro CF, Kotter MR. A Novel Insight Into the Challenges of Diagnosing Degenerative Cervical Myelopathy Using Web-Based Symptom Checkers. J Med Internet Res 2019 11;21(1):e10868. PMID:30300137

22. Fehlings MG, Tetreault LA, Riew KD, Middleton JW, Aarabi B, Arnold PM, Brodke DS, Burns AS, Carette S, Chen R, Chiba K, Dettori JR, Furlan JC, Harrop JS, Holly LT, Kalsi-Ryan S, Kotter M, Kwon BK, Martin AR, Milligan J, Nakashima H, Nagoshi N, Rhee J, Singh A, Skelly AC, Sodhi S, Wilson JR, Yee A, Wang JC. A Clinical Practice Guideline for the Management of Patients With Degenerative Cervical Myelopathy: Recommendations for Patients With Mild, Moderate, and Severe Disease and Nonmyelopathic Patients With Evidence of Cord Compression. Glob Spine J 2017 Sep 1;7(3_suppl):70S-83S. [doi: 10.1177/2192568217701914]

23. Fehlings MG, Ibrahim A, Tetreault L, Albanese V, Alvarado M, Arnold P, Barbagallo G, Bartels R, Bolger C, Defino H, Kale S, Massicotte E, Moraes O, Scerrati M, Tan G, Tanaka M, Toyone T, Yukawa Y, Zhou Q, Zileli M, Kopjar B. A global perspective on the outcomes of surgical decompression in patients with cervical spondylotic myelopathy: results from the prospective multicenter AOSpine international study on 479 patients. Spine 2015 Sep 1;40(17):1322–1328. PMID:26020847

24. Fehlings MG, Wilson JR, Kopjar B, Yoon ST, Arnold PM, Massicotte EM, Vaccaro AR, Brodke DS, Shaffrey CI, Smith JS, Woodard EJ, Banco RJ, Chapman JR, Janssen ME, Bono CM, Sasso RC, Dekutoski MB, Gokaslan ZL. Efficacy and safety of surgical decompression in patients with cervical spondylotic myelopathy: results of the AOSpine North America prospective multi-center study. J Bone Joint Surg Am 2013 Sep 18;95(18):1651–1658. PMID:24048552

25. Mowforth OD, Davies BM, Goh S, O'Neill CP, Kotter MRN. Research Inefficiency in Degenerative Cervical Myelopathy: Findings of a Systematic Review on Research

For peer review only

Activity Over the Past 20 Years. Glob Spine J 2019 Jun 12;2192568219847439. [doi: 10.1177/2192568219847439]

26. Davies BM, Khan DZ, Mowforth OD, McNair AGK, Gronlund T, Kolias AG, Tetreault L, Starkey ML, Sadler I, Sarewitz E, Houlton D, Carter J, Kalsi-Ryan S, Aarabi B, Kwon BK, Kurpad SN, Harrop J, Wilson JR, Grossman R, Curt A, Fehlings MG, Kotter MRN. RE-CODE DCM (REsearch Objectives and Common Data Elements for Degenerative Cervical Myelopathy): A Consensus Process to Improve Research Efficiency in DCM, Through Establishment of a Standardized Dataset for Clinical Research and the Definition of the Research Priorities. Glob Spine J 2019 May;9(1 Suppl):65S-76S. PMID:31157148

27. Kalsi-Ryan S, Karadimas SK, Fehlings MG. Cervical Spondylotic Myelopathy The Clinical Phenomenon and the Current Pathobiology of an Increasingly Prevalent and Devastating Disorder. The Neuroscientist 2013 Aug 1;19(4):409–421. PMID:23204243

28. Fehlings MG, Wilson JR, Karadimas SK, Arnold PM, Kopjar B. Clinical evaluation of a neuroprotective drug in patients with cervical spondylotic myelopathy undergoing surgical treatment: design and rationale for the CSM-Protect trial. Spine 2013 Oct 15;38(22 Suppl 1):S68-75. PMID:23962993

29. Kato S, Fehlings M. Degenerative cervical myelopathy. Curr Rev Musculoskelet Med 2016 Jun 1;9(3):263–271. PMID:27250040

30. Morris ZS, Wooding S, Grant J. The answer is 17 years, what is the question: understanding time lags in translational research. J R Soc Med 2011 Dec;104(12):510–520. PMID:22179294

31. Pokryszko-Dragan A, Mottershead J, Aitken G. Attitudes towards neurology among medical undergraduates. Neurol Neurochir Pol 2019;53(1):61–73. [doi: 10.5603/PJNNS.a2018.0004]

32. Shiels L, Majmundar P, Zywot A, Sobotka J, Lau CSM, Jalonen TO. Medical student attitudes and educational interventions to prevent neurophobia: a longitudinal study. BMC Med Educ 2017 Nov 21;17(1):225. PMID:29162061

33. Conway S, Tubridy N. "Neurophobia": More Nurture than Nature? Ir Med J 2018 14;111(3):710. PMID:30376228

34. King CJ, Bolton A, Guerrasio J, Trosterman A. Defining Medical Student Patient Care Responsibilities Before Intern Year: Results of a National Survey. South Med J 2017;110(12):765–769. PMID:29197310

35. Edwards PJ, Roberts I, Clarke MJ, Diguiseppi C, Wentz R, Kwan I, Cooper R, Felix LM, Pratap S. Methods to increase response to postal and electronic questionnaires. Cochrane Database Syst Rev 2009 Jul 8;(3):MR000008. PMID:19588449

For peer review only

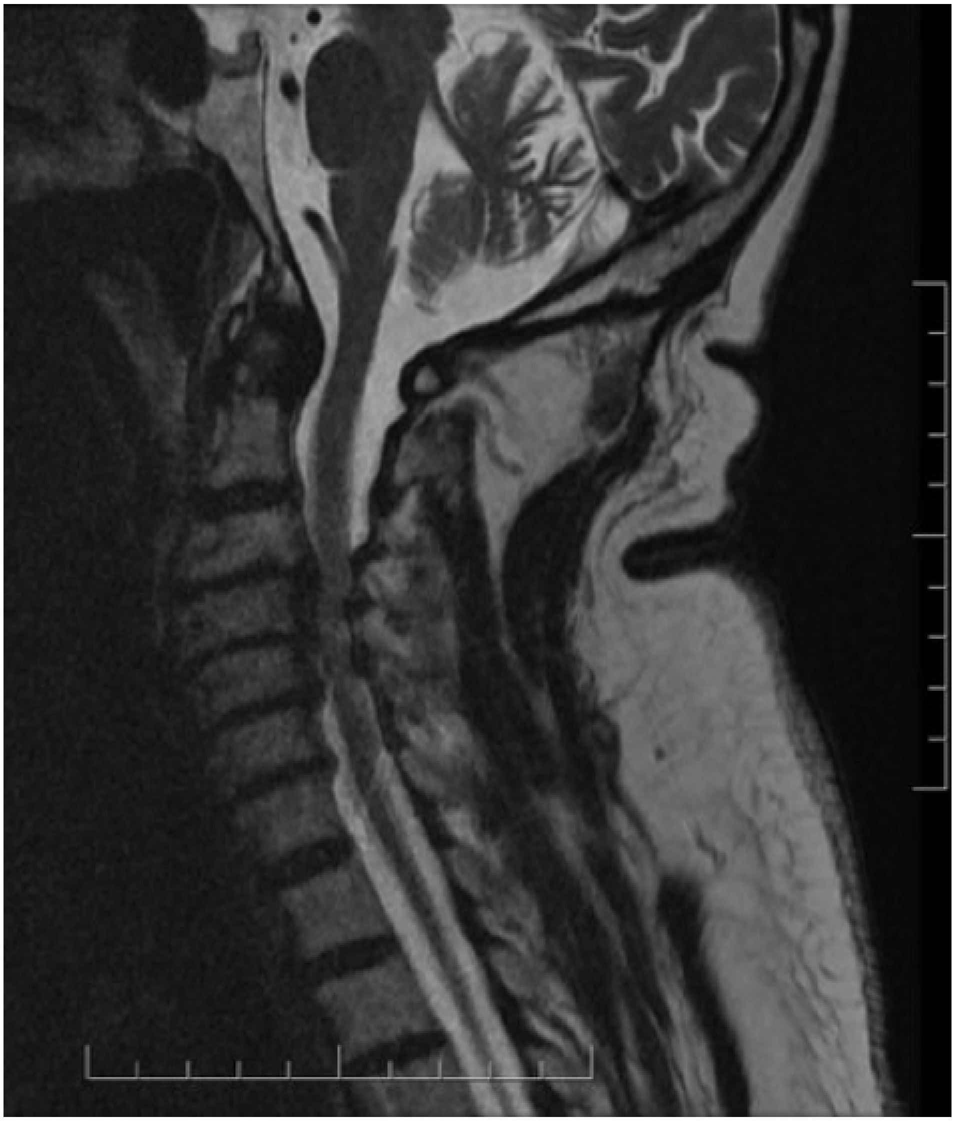

Figure 1. Typical MRI scan from patient with DCM showing spinal cord compression and multi-level degenerative changes.

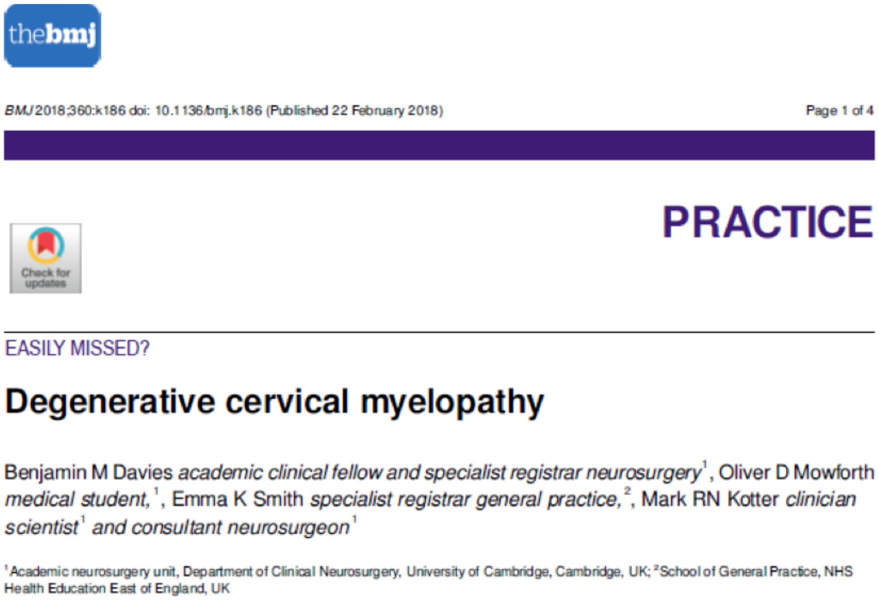
thebmj

BMJ 2018;360:k186 doi: 10.1136/bmj.k186 (Published 22 February 2018) Page 1 of 4

PRACTICE

Check for updates

EASILY MISSED?

Degenerative cervical myelopathy

Benjamin M Davies *academic clinical fellow and specialist registrar neurosurgery*[1], Oliver D Mowforth *medical student,*[1], Emma K Smith *specialist registrar general practice,*[2], Mark RN Kotter *clinician scientist*[1] *and consultant neurosurgeon*[1]

[1]Academic neurosurgery unit, Department of Clinical Neurosurgery, University of Cambridge, Cambridge, UK; [2]School of General Practice, NHS Health Education East of England, UK

Figure 2. DCM Educational Article commissioned by the British Medical Journal.

MYELOPATHY.ORG STUDENT SOCIETY

Myelopathy Student Survey

First Question. Consent: I have read the participant information sheet and consent to my survey response being used in this study (yes/no).

Survey Part 1: Teaching

- Which medical school do you study at?
  (dropdown selection of all UK medical schools, including other category)
- Preclinical or clinical?
  (dropdown selection of preclinical or clinical)
- Which year of medical school?
  (dropdown selection: year1; year2; year3; year4; year5; year6; other)
- What is your planned/most likely career speciality?
  (free entry)
- Have you heard of degenerative cervical myelopathy (also known as cervical spondylotic myelopathy)?
  (dropdown selection: yes; no)
- Have you had any teaching on cervical myelopathy from your medical school?
  (dropdown selection: yes; no)
- What is the total duration of your cervical myelopathy teaching?
  (dropdown selection: 0 mins; 15 mins; 30 mins; 1 hour; 2 hours; >2 hours)
- What form did the teaching take?
  (dropdown selection: lecture; seminar; ward-based teaching; e-learning; other; N/A)
- How much private study have you done on cervical myelopathy?
  (dropdown selection: 0 mins; 15 minutes; 30 minutes; 1 hour; 2 hours; >2 hours)

Survey Part 2: Knowledge

For many of the following questions there is often not necessarily a right or wrong and we are often more interested in your perception.

- How would you rate your overall knowledge of cervical myelopathy?
  (dropdown selection: excellent, very good, average, poor, very poor)
- What do you estimate is the prevalence of cervical myelopathy is in the over 40s?
  (dropdown selection: 0.01%; 0.1%; 1%; 5%; 10%; 50%)
- Which do you think is the best imaging modality to diagnose myelopathy? (4 or 5 choices)
  (dropdown selection: MRI; CT; X-ray; myelography; ultrasound)
- What do you think the average time to diagnosis is from first presentation? (5 choices)
  (dropdown selection: 1 week; 1 month; 1 year; 2 years; 5 years)
- On average, how many consultations do you think there are before a patient is diagnosed?
  (dropdown selection 1;2;5;10;20)
- Do you think time to diagnosis is likely important for long-term prognosis?
  (dropdown selection: yes; no)
- What effect do you think surgery is likely to have on a patient's symptoms?
  (dropdown selection: improve; stabilise; worsen)
- Which of these symptoms might be present in cervical myelopathy?
  (clumsy gait; neck pain/stiffness; falls; imbalance; loss of bowel control; loss of urinary control; erectile dysfunction; paraesthesia; limb weakness; limb numbness; limb pain; loss of dexterity)
- Please rank these conditions from worst to best in terms of your estimate of patient quality of life?
  (dropdown selection: cancer; cervical myelopathy; diabetes; depression; heart failure)
- How would you most like to learn about cervical myelopathy in the future?
  (dropdown selection: lecture; seminar; private study; online e-learning material; not interested)

Figure 3. Final Survey Design

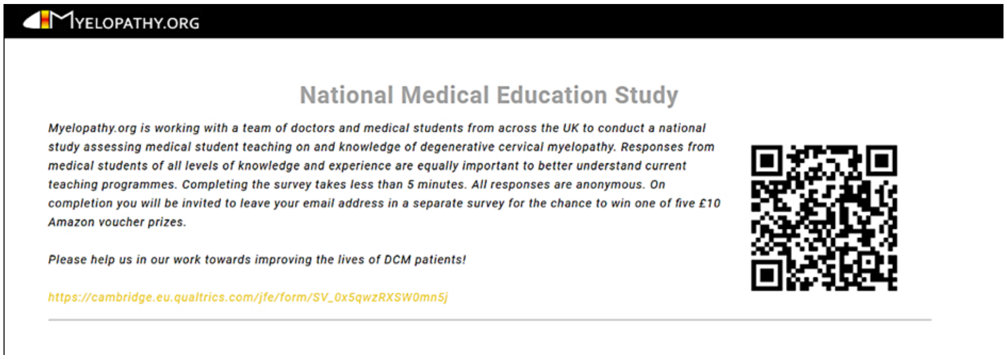

Figure 4. A standard advertisement devised by the protocol development team and approved by the research ethics committee is being used for all survey advertising.

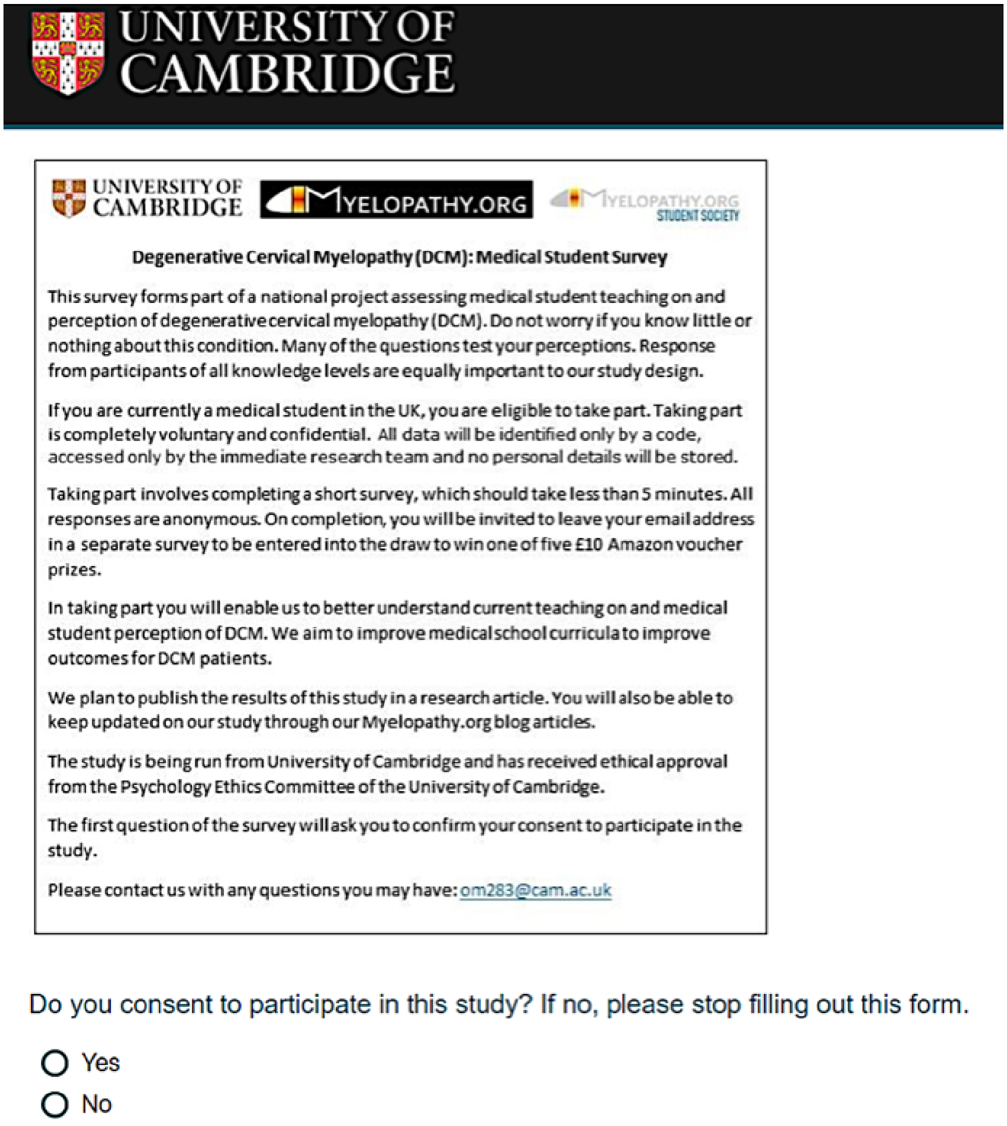

UNIVERSITY OF CAMBRIDGE

UNIVERSITY OF CAMBRIDGE MYELOPATHY.ORG MYELOPATHY.ORG STUDENT SOCIETY

Degenerative Cervical Myelopathy (DCM): Medical Student Survey

This survey forms part of a national project assessing medical student teaching on and perception of degenerative cervical myelopathy (DCM). Do not worry if you know little or nothing about this condition. Many of the questions test your perceptions. Response from participants of all knowledge levels are equally important to our study design.

If you are currently a medical student in the UK, you are eligible to take part. Taking part is completely voluntary and confidential. All data will be identified only by a code, accessed only by the immediate research team and no personal details will be stored.

Taking part involves completing a short survey, which should take less than 5 minutes. All responses are anonymous. On completion, you will be invited to leave your email address in a separate survey to be entered into the draw to win one of five £10 Amazon voucher prizes.

In taking part you will enable us to better understand current teaching on and medical student perception of DCM. We aim to improve medical school curricula to improve outcomes for DCM patients.

We plan to publish the results of this study in a research article. You will also be able to keep updated on our study through our Myelopathy.org blog articles.

The study is being run from University of Cambridge and has received ethical approval from the Psychology Ethics Committee of the University of Cambridge.

The first question of the survey will ask you to confirm your consent to participate in the study.

Please contact us with any questions you may have: om283@cam.ac.uk

Do you consent to participate in this study? If no, please stop filling out this form.

- Yes
- No

Figure 5. Survey Front Page including Patient Information Sheet and Capturing of Informed Consent.

Current Provision of Myelopathy Education in Medical Schools in the United Kingdom: Protocol for a National Medical Student Survey

Journal:	*BMJ Open*
Manuscript ID	bmjopen-2019-035563.R1
Article Type:	Protocol
Date Submitted by the Author:	20-Mar-2020
Complete List of Authors:	Mowforth, Oliver; University of Cambridge, Division of Neurosurgery, Department of Clinical Neurosciences<br>Davies, Benjamin; University of Cambridge, Division of Neurosurgery, Department of Clinical Neurosciences<br>Stewart, Max; University of Cambridge, School of Clinical Medicine<br>Smith, Sam; University of Cambridge, School of Clinical Medicine<br>Willison, Alice; University of Dundee, Ninewells Hospital and Medical School<br>Ahmed, Shahzaib; University of Cambridge, School of Clinical Medicine<br>Starkey, Michelle; Myelopathy.org<br>Sadler, Iwan; Myelopathy.org<br>Sarewitz, Ellen; Myelopathy.org<br>Stacpoole, Sybil; University of Cambridge, Neurology Unit, Department of Clinical Neurosciences<br>Kotter, Mark; University of Cambridge, Department of Clinical Neurosurgery; Ann McLaren Laboratory of Regenerative Medicine, Department of Clinical Neurosciences
**Primary Subject Heading**:	Neurology
Secondary Subject Heading:	Surgery
Keywords:	NEUROSURGERY, Neurosurgery < SURGERY, MEDICAL EDUCATION & TRAINING, NEUROLOGY

I, the Submitting Author has the right to grant and does grant on behalf of all authors of the Work (as defined in the below author licence), an exclusive licence and/or a non-exclusive licence for contributions from authors who are: i) UK Crown employees; ii) where BMJ has agreed a CC-BY licence shall apply, and/or iii) in accordance with the terms applicable for US Federal Government officers or employees acting as part of their official duties; on a worldwide, perpetual, irrevocable, royalty-free basis to BMJ Publishing Group Ltd ("BMJ") its licensees and where the relevant Journal is co-owned by BMJ to the co-owners of the Journal, to publish the Work in this journal and any other BMJ products and to exploit all rights, as set out in our licence.

The Submitting Author accepts and understands that any supply made under these terms is made by BMJ to the Submitting Author unless you are acting as an employee on behalf of your employer or a postgraduate student of an affiliated institution which is paying any applicable article publishing charge ("APC") for Open Access articles. Where the Submitting Author wishes to make the Work available on an Open Access basis (and intends to pay the relevant APC), the terms of reuse of such Open Access shall be governed by a Creative Commons licence – details of these licences and which Creative Commons licence will apply to this Work are set out in our licence referred to above.

Other than as permitted in any relevant BMJ Author's Self Archiving Policies, I confirm this Work has not been accepted for publication elsewhere, is not being considered for publication elsewhere and does not duplicate material already published. I confirm all authors consent to publication of this Work and authorise the granting of this licence.

For peer review only

Current Provision of Myelopathy Education in Medical Schools in the United Kingdom: Protocol for a National Medical Student Survey

Oliver D. Mowforth B.A. M.B. B.Chir,[1] Benjamin M. Davies B.Sc. MRCS,[1] Max Stewart B.A,[2] Sam Smith B.A.,[2] Alice Willison B.Sc. MRes MBChB,[3], Shahzaib Ahmed B.A.,[2] Michelle L. Starkey Ph.D.,[4] Iwan Sadler,[4] Ellen Sarewitz M.A.,[4] Sybil R. Stacpoole Ph.D.,[5], Mark R. Kotter M.D. M.Phil Ph.D.[1,6]

1. Division of Neurosurgery, Department of Clinical Neurosciences, University of Cambridge, Cambridge, UK
2. School of Clinical Medicine, University of Cambridge, Cambridge, UK
3. Ninewells Hospital and Medical School, University of Dundee, Dundee, UK
4. Myelopathy.org, Cambridge, UK
5. Neurology Unit, Department of Clinical Neurosciences, University of Cambridge, Cambridge, UK
6. Anne McLaren Laboratory for Regenerative Medicine, Wellcome Trust-Medical Research Council Cambridge Stem Cell Institute, University of Cambridge, Cambridge, UK.

Key words: spinal cord diseases; cervical vertebrae; spondylosis; spinal osteophytosis; surveys and questionnaires; students, medical; education, medical

Corresponding author:

Dr Oliver Mowforth
Division of Neurosurgery
Department of Clinical Neurosciences
University of Cambridge
CB2 0SZ
Email: omowforth@gmail.com
Telephone: +44 1223 763366
Fax: +44 1223 763350

Reference count: 35

Full text word count: 2550

Figure count: 5

For peer review only

Abstract

Introduction

Degenerative cervical myelopathy (DCM) is a common, disabling and progressive neurological condition triggered by chronic compression of the cervical spinal cord by surrounding degenerative changes. Early diagnosis and specialist management are essential to reduce disability, yet time to diagnosis is typically prolonged. Lack of sufficient representation of DCM in undergraduate and postgraduate medical curricula may contribute to the poor recognition of DCM by non-specialist doctors in clinical practice.

In this study our objective therefore is to assess DCM teaching provision in medical schools throughout the United Kingdom (UK) and to assess the impact of teaching on the DCM knowledge of UK medical students.

Methods

A 19-item questionnaire capturing data on medical student demographics, myelopathy teaching and myelopathy knowledge was designed. Ethical approval was granted by the Psychology Research Ethics Committee, University of Cambridge. An online survey was hosted on Myelopathy.org, an international myelopathy charity. Students studying at a UK medical school are eligible for inclusion. The survey is advertised nationally through university social media pages, university email bulletins and the national student network of Myelopathy.org. Advertisements are scheduled monthly over a 12-month recruitment period. Participation is incentivised by entering consenting participants of completed surveys to an Amazon voucher prize draw. Responses are anonymised using participant-chosen unique identifier codes. A participant information sheet followed by an explicit survey question captured participant informed consent. The study is due to report in Summer 2020. Regular updates on the progress of the study will be published on Myelopathy.org.

Ethics and Dissemination

For peer review only

Ethical Approval for the study was granted by the Psychology Research Ethics Committee, University of Cambridge (PRE.2018.099). Insurance cover was arranged via the University of Cambridge (HVS/2018/2366). The findings of the study described in this protocol, and all other related work, will be submitted for publication in a peer-reviewed journal and will be presented at scientific conferences.

For peer review only

Strengths and limitations of this study

- This is a large national questionnaire-based study targeted at all medical students in the United Kingdom
- This is the first study to assess DCM medical education in the UK
- This is a student-led study with oversight from senior researchers and involvement of DCM patients
- The study data will provide insight into an area that has potential to translate into substantial improvements in patient outcomes
- Risk of selection bias may overestimate the current knowledge of DCM among UK medical students

For peer review only

Introduction

Degenerative cervical myelopathy (DCM) is a common neurological condition of symptomatic cervical spinal cord compression due to degenerative changes of the spine (Figure 1).[1] DCM is the most common cause of spinal cord impairment in adults worldwide, with an estimated prevalence of up to 5% in the over 40s.[1],[2],[3].

Substantial improvements in the care of DCM patients could be realised today without any additional scientific understanding of the condition.[1] DCM patients almost universally experience diagnostic delays. For example, one study demonstrated that on average it took 2 years and 5 clinic appointments before the diagnosis was made.[4] Improvement in diagnosis is therefore a key target which would provide immediate reductions in disability.[5]

Initially, DCM patients are commonly seen by primary care, emergency and medical physicians.[6],[7] These non-specialist triage points appear key to earlier diagnosis, yet DCM features poorly in medical curricula.[8] This risks non-specialist doctors being poorly equipped to detect DCM.

The current focus and makeup of DCM medical education is unclear. Our recent evaluation of undergraduate and post-graduate medical curricula identified deficiencies in DCM representation, however collectively this did not correlate with student performance in multiple-choice question banks.[8]

The aim of this study is to describe the teaching of DCM in UK medical schools and to assess the influence on medical student knowledge of DCM. Ultimately, the aim is to identify both training and knowledge gaps to inform guidance on DCM education in order to address the burden and impact of delayed diagnosis. We hypothesise that knowledge and awareness amongst medical professionals is related to their specific medical education on DCM.

For peer review only

Methods

Study Partners

Myelopathy.org

Myelopathy.org is an international cervical myelopathy charity, which aims to improve health and well-being in DCM.[9] This study forms part of a wider project to evaluate DCM education and awareness amongst medical professionals in the UK, and develop focused and effective interventions to improve this. This includes our recent gap-analysis of key medical resources and knowledge at each stage of the primary care training pathway[10] and an educational article on DCM for non-specialist medical professionals, commissioned by the British Medical Journal (Figure 2).[1] The charity is focussed on understanding how medical education can be improved as one possible strategy to address the current problems of delayed, missed and under-diagnosis.[1]

Student Society of Myelopathy.org

The foundations of professional knowledge begin with students' formative experiences at medical school. Therefore, the Student Society of Myelopathy.org was established in 2018 with the aim of improving DCM engagement and awareness amongst medical students. The society remains in its infancy but has hosted a series of educational talks at the University of Cambridge; established national medical student essay and research prizes; designed and initiated this current study; and secured sponsorship from corporate partners to support these projects.

Study Design

An electronic survey was agreed to be the most efficient way to simultaneously assess teaching, perceptions of teaching, and current knowledge, and to efficiently reach students from all UK medical schools. Part One of the survey was designed to capture data on basic demographic characteristics and teaching of DCM in UK medical schools. Part Two was designed to capture data on knowledge and awareness of fundamental DCM facts including prevalence, symptoms, diagnostic imaging modalities and surgical effectiveness in addition

For peer review only

to appreciation of important current challenges such as prolonged time to diagnosis and poor patient quality of life. Part Two was designed to capture participant perception of their DCM knowledge. These questions were considered to be a reasonable balance of capturing sufficient data whilst maintaining an acceptably short survey completion time of approximately 5 minutes.

The study management team comprised representation from second year to final year students from multiple UK medical schools. The team comprised an academic neurosurgical registrar [BMD] and an academic neurosurgical consultant [MRK], both with special clinical and academic interests in DCM, and an academic consultant neurologist [SRS] who is a neuroscience teaching fellow and neuroscience course organiser at the University of Cambridge. Importantly, this is a student-motivated, student-led project with supervision, oversight and mentorship at all stages of the study from a team of senior researchers.

The survey was iteratively refined amongst the study management group until section one was felt to capture sufficient information on current DCM teaching provision in UK medical schools. Similarly, section two was refined until it was felt to evaluate fundamental DCM facts at a level sufficient for a non-specialist, but qualified doctor to appropriately suspect, investigate, diagnose and re-direct care in DCM. In total, a 19-item questionnaire was developed (Figure 3).

Survey development closely aligns with the AMEE 7 step guide to medical education survey development: (1) literature review; (2) focus group establishment (3) synthesis of literature review/focus group (4) item development (5) expert validation (6) feedback and expert validation and (7) piloting, [11] with the exception that a quantitative gap analysis was performed in the place of a literature review due to the lack of research on DCM medical education at the time of study design.[8] Nonetheless, planning for a systematic review of similar medical education surveys in other diseases is underway.

Survey Piloting

The survey was piloted in January 2019 by a group of 20 medical students at the University of Cambridge. These students were not directly involved in the design of the study. Data were inspected to identify any inconsistencies, ambiguities and problematic survey items. No issues were identified. Students in the pilot group were contacted to ask if they had encountered any difficulties, areas of uncertainty or had suggestions for improving the survey Students reported the survey to be acceptable on both desktop and mobile devices. Informal

For peer review only - http://bmjopen.bmj.com/site/about/guidelines.xhtml

cognitive interviews of 5 students revealed that survey items were interpreted by students as intended by the study team, without ambiguity. No further alterations were therefore made. No formal statistical data analysis was conducted at the pilot stage.

Survey Administration

To maximise reach across the UK, the survey was hosted on Qualtrics Survey Software (Provo, United States), a professional online survey platform. Qualtrics Survey Software includes both desktop and mobile-compatible versions, maximising convenience for participants.

The survey comprises two pages. The first page acts as a front page consisting of a participant information sheet and a question capturing informed consent. The second page is the main survey page. It comprises 19 questions over a single page to minimise the numbers of clicks required for participants to complete the survey.

Survey Dissemination

A network of Myelopathy.org Student Representatives was recruited, with representation of students from 25 different medical schools across the UK. Some but not all were also representatives for the Society of British Neurological Surgeons (SBNS) Neurology and Neurosurgery Student Interest Group (NANSIG). A standard advertisement devised by the protocol development team and approved by the research ethics committee is utilised for all advertising (Figure 4). Representatives from all medical schools are sent monthly reminder emails to disseminate adverts at their institution over a 12-month period from February 2019 – January 2020. There was no difference in the timing of reminder emails between different medical schools. Although prompted to advertise monthly, the final decision on frequency of advertising at each institution is left to the discretion of representatives, guided by local rules.

On completion of the survey participants are invited to leave an email address in a separate Google Forms survey to be entered into a prize draw to win one of five £10 Amazon vouchers. The prize draw was agreed by the study management group and approved by the research ethics committee. The rationale was that prizes of this value may encourage participation without providing an inappropriate incentive to participate.

The survey is advertised in social media groups and on social media pages for relevant student groups and societies at universities across the UK. For example, Facebook groups of surgical, neurosurgical, neurological and other medical student societies were approached. In

For peer review only

addition, university mailing lists and email bulletins are being utilised, where appropriate, in accordance with local rules. Adverts are also placed in the monthly NANSIG newsletter, emailed out to medical student subscribers across the UK. The survey link is also hosted on the Student Society of Myelopathy.org webpage and Facebook page.

Eligibility and Representation

All UK medical students are eligible to complete the survey. We aim to secure representation of all UK medical schools.

Consent and Confidentiality

A patient information sheet (PIS) including a question capturing informed consent forms the survey frontpage (Figure 5). The PIS emphasises the rational, purpose, study aims and the voluntariness of participation. By necessity the background information on DCM in the PIS is minimal to avoid compromising the DCM knowledge-based questions in the survey. A page block is utilised to separate the front and main survey pages.

Participants assign themselves a unique identifier based on a defined combination of characters from their mother's maiden name, the street they grew up on and their mobile phone number; this ensures anonymity and confidentiality whilst allowing the possibility of linking together future surveys from the same participant.

Data Security

All data are stored exclusively on the secure, online Qualtrics platform until closure of the survey. Thereafter, survey data will be extracted directly from the Qualtrics platform into a password-encrypted Excel spreadsheet (Microsoft, California) on a password-protected computer. Only the immediate research team will be granted access to this on a need-to-access basis. After completion of data analysis, all data that is no longer required will be deleted. No participant-identifiable data will be collected or stored at any point.

Statistical Analysis

Data will be analysed in Microsoft Excel and using statistical software.

Relationships between duration of DCM teaching, duration of private study on DCM and student perception of their DCM knowledge and objective performance in the DCM knowledge-based questions will be assessed. Differences in performance on the knowledge-based questions will be compared between groups based on medical school, year of medical

For peer review only

school, duration of teaching, format of teaching, duration of private study, prior self-reported awareness of DCM, self-rating of DCM knowledge and between groups of medical students wishing to pursue neurological/neurosurgical career specialties and those who do not.

Due to all items in the survey being mandatory, we anticipate that missing data will be minimal, however will conduct a missing data analysis as necessary. It is not expected the data will be suitable for imputation, thus we will compare demographics to establish any selection bias caused by the exclusion of missing values. We will perform analysis on all collected values. The survey findings will be presented using descriptive statistics. Inferential statistical tests may be used to consider the relationship between knowledge and educational provision. Distribution testing will be performed. Appropriate statistical methods will be selected based on the class and distribution of the data; it is anticipated data will be non-parametric, and as measures are recorded on an ordinal or categorial basis, will therefore be compared using tests such as Chi-Squared or Kruksall Wallis Tests.

Patient and Public Involvement

Patients were involved in the conception, design, development and conduct of this study. A DCM Patient and Public Engagement day hosted at the University of Cambridge was captured by Cambridge TV in a documentary.[12] During this day, a focus group of DCM patients emphasised their experiences of diagnostic delays. This has been frequently echoed by patients in Myelopathy.org social media groups and in a DCM word-cloud creating initiative to understand patient's perceptions of the condition for a related project. DCM patients therefore inspired the conception of this study and approved the final survey design. Patients also assisted in the essential administrative tasks of the project, such as logo design. The online survey for the study was hosted on Myelopathy.org, which is a patient-maintained website for people with DCM. Patients were, therefore, active in aiding the dissemination of the survey to UK medical students. Patients who were involved in preparation of the manuscript are among the authors. DCM patients are involved in plans to disseminate this research to the patient community, including blog articles on Myelopathy.org, posts in online patient support groups and presence at spinal conferences in the UK.

For peer

Discussion

Study Rationale

This is the first study to describe the DCM education at medical schools nationally. Medical students are recruited to an online survey, including an assessment of their DCM knowledge and their personal DCM learning experience. By comparison of learning experience and knowledge, and also comparison between medical schools, it is anticipated both the presence or absence of a knowledge gap can be evaluated, and the more successful teaching formats identified. This will be used to inform Myelopathy.org education recommendations.

Why assess DCM education?

DCM is a devastating condition with one of the poorest quality of life scores of any chronic disease.[13] Symptoms include loss of manual dexterity, gait impairment, limb paraesthesia, limb weakness, limb stiffness, bladder and bowel disturbance, neck pain and neck stiffness.[1],[14],[15] The full spectrum of symptoms and the natural history of DCM is poorly appreciated,[16], [17] yet it remains clear that the condition poses a substantial burden on the physical and mental health of people with DCM [18] and their supporters [19]. There is a broad differential for DCM symptoms[20] and poor consensus on what the most common DCM symptoms are. In addition, an increasing number of atypical symptoms, such as dysaesthetic sensory symptoms,[6] chest tightness,[21] and headache[22] are being reported.[23] Neurological examination findings include upper motor neuron signs in the upper and lower limbs: spasticity and hyperreflexia in addition to weakness.[1],[15].

The latest international guidelines[24] recommend surgical decompression as the only evidence-based management [25],[26] for patients with moderate to severe, or progressive DCM. Surgery is also a management option in patients with mild DCM.[24] Importantly, surgery can halt progression of the condition but is ineffective at reversing existing damage.[26] Therefore, the traditional medical dogma that prevention is better than cure holds as strongly as ever in DCM: time is spine.[1]

Scientific advances are sorely needed to improve therapeutic options and ultimately to cure disabled patients with DCM.[27], [28] However, whilst research into the neurobiology of DCM and neural regeneration,[29] such as the RECEDE-MYELOPATHY and CSM-PROTECT trials offer hope for the future, [9],[30],[31] even the most optimistic scientist

For peer review only

would concede that tangible benefits for patients arising from these technologies remain on the horizon: one systematic review reported average duration of knowledge translation of 17 years from research to practice.[32]

Therefore, pragmatic approaches are required to improve outcomes for today's DCM patients. One such approach entails addressing the substantial problem of diagnostic delays[1], which is expected to improve outcomes in this progressive condition.[5] One method of instituting this may be through better education. Indeed, a phenomenon of "neurophobia" is well-described amongst medical students and non-specialist doctors.[33],[34],[35] Additionally, evidence from our recent gap analysis suggests poor representation of DCM in medical curricula.[10] These factors may point to a knowledge gap in DCM medical education.

Benefits of a medical student survey

Assessing teaching and knowledge among medical students is a pragmatic and initial step to evaluate a knowledge gap. Medical school is the foundation of all medical professionals' education and is especially important for instilling basic knowledge of important areas of medicine that may fall outside a doctor's future field of specialisation. It is likely that this is particularly pertinent for knowledge that this not later core (i.e. built on or refined) during specialisation. For this purpose, a survey of medical students is best placed to simultaneously assess knowledge, whilst reporting on the current provision of DCM teaching and perceptions of it. Moreover, the survey itself may help to prime an audience to support a subsequent intervention, or at the very least raise awareness.

Limitations

Online surveys are an effective method of reaching students from diverse medical schools.[36] In addition, digital collection of data offers a secure and seamless stream, facilitating data analysis. However, their limitation is in understanding how representative the findings are.

In order to mitigate this, a number of different dissemination approaches are being employed. This includes the appointment of local representatives at each UK medical school, the use social media posts, society mailing lists; society newsletters; medical school email bulletins; medical school newsletters, and also the use of prizes as an incentive.[37] Additionally, the capture of participant demographics will help to measure the sample characteristics.

For peer review only

The engagement with open surveys, often involves some predefined interest. It is possible therefore a selection-bias towards students interested in neuroscience is more likely and results would be considered as a best-case scenario. Whilst there is a risk of overlooking or underestimating the magnitude of a knowledge gap given the outlined benefits of this methodology, this is an accepted risk. Whatever the relationship between DCM teaching and knowledge turns out to be, it will be instructive with regards to the overall objective of identifying ways of improving DCM diagnosis.

We acknowledge that wording of the survey item “Do you think time to diagnosis is important for long term prognosis” could be perceived a leading and would have been better phrased in a more neutral format such as “How important is time to diagnosis for long-term prognosis?”. We will monitor the performance of this item closely in our analysis.

Ethics and Dissemination

Ethical Approval for the study was granted by the Psychology Research Ethics Committee, University of Cambridge (PRE.2018.099). Insurance cover was arranged via the University of Cambridge (HVS/2018/2366). The findings of the study described in this protocol, and all other related work, will be submitted for publication in a peer-reviewed journal and will be presented at scientific conferences.

Conclusion

Urgent improvements in outcomes for DCM patients are needed. Earlier diagnosis would improve outcomes based on current treatments. This study aims to evaluate DCM education and knowledge, and explore their relationship, amongst medical students. It is hoped this will inform educational interventions that may offer the greatest chance of shaping the non-specialist doctor’s diagnosis of DCM tomorrow.

For peer review only

Author contributions:

ODM, BMD, IS, ES, SRS and MRK were involved in the conceptualisation and design of this study. ODM wrote the first draft of the manuscript. ODM, BMD, MS, SS, AW, SA, MLS, IS, ES, SRS and MRK reviewed and approved the final manuscript.

Funding:

Research in the MRNK's laboratory is supported by a core support grant from the Wellcome Trust and MRC to the Wellcome Trust-Medical Research Council Cambridge Stem Cell Institute. MRNK is supported by a NIHR Clinician Scientist Award and BMD is supported by a NIHR Clinical Doctoral Research Fellowship.

Disclaimer:

This report is independent research arising from a Clinician Scientist Award, CS-2015-15-023, supported by the National Institute for Health Research. The views expressed in this publication are those of the authors and not necessarily those of the NHS, the National Institute for Health Research or the Department of Health and Social Care.

Presented at:

World Federation of Neurosurgical Societies International Meeting, Belgrade, Serbia, March 2019.

British Neurosurgical Research Group Meeting, Edinburgh, November 2019

Competing Interests:

ODM, BMD, MS, SS, AW, SA, MLS, IS, ES and MRK have voluntary roles at Myelopathy.org, an international DCM charity.

Patient consent for publication: not required

Ethical approval: approved by Psychology Research Ethics Committee, University of Cambridge

Data availability statement: All data relevant to the study are included in the article or uploaded as online supplementary information.

For peer review only

Acknowledgements:

The authors are grateful to all the patients who have inspired this study, most especially at the University of Cambridge Patient and Public Engagement Day. In addition, the authors would like to thank the Student Society of Myelopathy.org and NANSIG medical student representatives for their assistance in disseminating the research survey.

For peer review only

Figure Legends:

Figure 1. Typical MRI scan from patient with DCM showing spinal cord compression and multi-level degenerative changes.

Figure 2. DCM Educational Article commissioned by the British Medical Journal.

Figure 2. Final Survey Design

Figure 4. A standard advertisement devised by the protocol development team and approved by the research ethics committee is being used for all survey advertising.

Figure 5. Survey Front Page including Patient Information Sheet and Capturing of Informed Consent.

For peer review only

References

1. Davies BM, Mowforth OD, Smith EK, Kotter MR. Degenerative cervical myelopathy. BMJ 2018 Feb 22;360:k186. PMID:29472200

2. Kovalova I, Kerkovsky M, Kadanka Z, Kadanka Z, Nemec M, Jurova B, Dusek L, Jarkovsky J, Bednarik J. Prevalence and Imaging Characteristics of Nonmyelopathic and Myelopathic Spondylotic Cervical Cord Compression: SPINE 2016 Dec;41(24):1908–1916. [doi: 10.1097/BRS.0000000000001842]

3. Nouri A, Tetreault L, Singh A, Karadimas SK, Fehlings MG. Degenerative Cervical Myelopathy: Epidemiology, Genetics, and Pathogenesis. Spine 2015 Jun 15;40(12):E675-693. PMID:25839387

4. Behrbalk E, Salame K, Regev GJ, Keynan O, Boszczyk B, Lidar Z. Delayed diagnosis of cervical spondylotic myelopathy by primary care physicians. Neurosurg Focus 2013 Jul;35(1):E1. PMID:23815245

5. Cates JR, Soriano MM. Cervical spondylotic myelopathy. J Manipulative Physiol Ther 1995 Sep;18(7):471–475. PMID:8568430

6. Mowforth OD, Davies BM, Kotter MR. “I am not delusional!” Sensory dysaesthesia secondary to degenerative cervical myelopathy. BMJ Case Rep 2019 Apr 11;12(4). PMID:30975783

7. Radcliff KE, Curry EP, Trimba R, Walker JB, Purtill JJ, Austin MS, Parvizi J, Vaccaro AR, Hilibrand AS, Albert TJ. High Incidence of Undiagnosed Cervical Myelopathy in Patients With Hip Fracture Compared With Controls. J Orthop Trauma 2016 Apr;30(4):189–193. PMID:26562581

8. Waqar M, Wilcock J, Garner J, Davies B, Kotter M. Quantitative analysis of medical students’ and physicians’ knowledge of degenerative cervical myelopathy. BMJ Open 2020 Jan 12;10(1):e028455. PMID:31932384

9. Neurology TL. A focus on patient outcomes in cervical myelopathy. Lancet Neurol 2019 Jul 1;18(7):615. PMID:31202463

10. Davies B, Wilcock J, Garner J, Waqar M, Kotter M. A quantitative analysis of medical students’ and physicians’ knowledge of degenerative cervical myelopathy. 2019 Jun 10 [cited 2019 Sep 15]; [doi: 10.17863/CAM.40662]

11. Developing questionnaires for educational research: AMEE Guide No. 87: Medical Teacher: Vol 36, No 6 [Internet]. [cited 2020 Mar 13]. Available from: https://www.tandfonline.com/doi/full/10.3109/0142159X.2014.889814

12. Roberts E. Elemental Ideas: Cervical Spondylotic Myelopathy. http://www.myelopathy.org/the-csm-documentary.html.

For peer review only

13. Oh T, Lafage R, Lafage V, Protopsaltis T, Challier V, Shaffrey C, Kim HJ, Arnold P, Chapman J, Schwab F, Massicotte E, Yoon T, Bess S, Fehlings M, Smith J, Ames C. Comparing Quality of Life in Cervical Spondylotic Myelopathy with Other Chronic Debilitating Diseases Using the SF-36 Survey. World Neurosurg 2017 Jan 5; PMID:28065875

14. Arnold JG. The clinical manifestations of spondylochondrosis (spondylosis) of the cervical spine. Ann Surg 1955 Jun;141(6):872–889. PMID:14377330

15. Brain WR, Northfield D, Wilkinson M. The neurological manifestations of cervical spondylosis. Brain J Neurol 1952 Jun;75(2):187–225. PMID:14934989

16. Badhiwala JH, Wilson JR. The Natural History of Degenerative Cervical Myelopathy. Neurosurg Clin N Am 2018 Jan;29(1):21–32. PMID:29173433

17. Clair SS, Bell GR. Natural History of Cervical Spondylotic Myelopathy. Semin Spine Surg 2007 Mar 1;19(1):2–5. [doi: 10.1053/j.semss.2007.01.005]

18. Stoffman MR, Roberts MS, King JT. Cervical spondylotic myelopathy, depression, and anxiety: a cohort analysis of 89 patients. Neurosurgery 2005 Aug;57(2):307–313; discussion 307-313. PMID:16094160

19. Mowforth OD, Davies B, Kotter M. Poor quality of life in degenerative cervical myelopathy carers (Preprint). 2019 Jul 25 [cited 2019 Oct 21]; [doi: 10.17863/CAM.42010]

20. Tracy JA, Bartleson JD. Cervical spondylotic myelopathy. The Neurologist 2010 May;16(3):176–187. PMID:20445427

21. Kobayashi H, Kikuchi S, Otani K, Sekiguchi M, Sekiguchi Y, Konno S. Development of a self-administered questionnaire to screen patients for cervical myelopathy. BMC Musculoskelet Disord 2010 Nov 22;11:268. PMID:21092213

22. Schrot RJ, Mathew JS, Li Y, Beckett L, Bae HW, Kim KD. Headache relief after anterior cervical discectomy: post hoc analysis of a randomized investigational device exemption trial. J Neurosurg Spine 2014 Aug;21(2):217–222. PMID:24836655

23. Davies BM, Munro CF, Kotter MR. A Novel Insight Into the Challenges of Diagnosing Degenerative Cervical Myelopathy Using Web-Based Symptom Checkers. J Med Internet Res 2019 11;21(1):e10868. PMID:30300137

24. Fehlings MG, Tetreault LA, Riew KD, Middleton JW, Aarabi B, Arnold PM, Brodke DS, Burns AS, Carette S, Chen R, Chiba K, Dettori JR, Furlan JC, Harrop JS, Holly LT, Kalsi-Ryan S, Kotter M, Kwon BK, Martin AR, Milligan J, Nakashima H, Nagoshi N, Rhee J, Singh A, Skelly AC, Sodhi S, Wilson JR, Yee A, Wang JC. A Clinical Practice Guideline for the Management of Patients With Degenerative Cervical Myelopathy: Recommendations for Patients With Mild, Moderate, and Severe Disease and Nonmyelopathic Patients With Evidence of Cord Compression. Glob Spine J 2017 Sep 1;7(3_suppl):70S-83S. [doi: 10.1177/2192568217701914]

25. Fehlings MG, Ibrahim A, Tetreault L, Albanese V, Alvarado M, Arnold P, Barbagallo G, Bartels R, Bolger C, Defino H, Kale S, Massicotte E, Moraes O, Scerrati M, Tan G,

For peer review only

Tanaka M, Toyone T, Yukawa Y, Zhou Q, Zileli M, Kopjar B. A global perspective on the outcomes of surgical decompression in patients with cervical spondylotic myelopathy: results from the prospective multicenter AOSpine international study on 479 patients. Spine 2015 Sep 1;40(17):1322–1328. PMID:26020847

26. Fehlings MG, Wilson JR, Kopjar B, Yoon ST, Arnold PM, Massicotte EM, Vaccaro AR, Brodke DS, Shaffrey CI, Smith JS, Woodard EJ, Banco RJ, Chapman JR, Janssen ME, Bono CM, Sasso RC, Dekutoski MB, Gokaslan ZL. Efficacy and safety of surgical decompression in patients with cervical spondylotic myelopathy: results of the AOSpine North America prospective multi-center study. J Bone Joint Surg Am 2013 Sep 18;95(18):1651–1658. PMID:24048552

27. Mowforth OD, Davies BM, Goh S, O'Neill CP, Kotter MRN. Research Inefficiency in Degenerative Cervical Myelopathy: Findings of a Systematic Review on Research Activity Over the Past 20 Years. Glob Spine J 2019 Jun 12;2192568219847439. [doi: 10.1177/2192568219847439]

28. Davies BM, Khan DZ, Mowforth OD, McNair AGK, Gronlund T, Kolias AG, Tetreault L, Starkey ML, Sadler I, Sarewitz E, Houlton D, Carter J, Kalsi-Ryan S, Aarabi B, Kwon BK, Kurpad SN, Harrop J, Wilson JR, Grossman R, Curt A, Fehlings MG, Kotter MRN. RE-CODE DCM (REsearch Objectives and Common Data Elements for Degenerative Cervical Myelopathy): A Consensus Process to Improve Research Efficiency in DCM, Through Establishment of a Standardized Dataset for Clinical Research and the Definition of the Research Priorities. Glob Spine J 2019 May;9(1 Suppl):65S-76S. PMID:31157148

29. Kalsi-Ryan S, Karadimas SK, Fehlings MG. Cervical Spondylotic Myelopathy The Clinical Phenomenon and the Current Pathobiology of an Increasingly Prevalent and Devastating Disorder. The Neuroscientist 2013 Aug 1;19(4):409–421. PMID:23204243

30. Fehlings MG, Wilson JR, Karadimas SK, Arnold PM, Kopjar B. Clinical evaluation of a neuroprotective drug in patients with cervical spondylotic myelopathy undergoing surgical treatment: design and rationale for the CSM-Protect trial. Spine 2013 Oct 15;38(22 Suppl 1):S68-75. PMID:23962993

31. Kato S, Fehlings M. Degenerative cervical myelopathy. Curr Rev Musculoskelet Med 2016 Jun 1;9(3):263–271. PMID:27250040

32. Morris ZS, Wooding S, Grant J. The answer is 17 years, what is the question: understanding time lags in translational research. J R Soc Med 2011 Dec;104(12):510–520. PMID:22179294

33. Pokryszko-Dragan A, Mottershead J, Aitken G. Attitudes towards neurology among medical undergraduates. Neurol Neurochir Pol 2019;53(1):61–73. [doi: 10.5603/PJNNS.a2018.0004]

34. Shiels L, Majmundar P, Zywot A, Sobotka J, Lau CSM, Jalonen TO. Medical student attitudes and educational interventions to prevent neurophobia: a longitudinal study. BMC Med Educ 2017 Nov 21;17(1):225. PMID:29162061

For peer review only

35. Conway S, Tubridy N. "Neurophobia": More Nurture than Nature? Ir Med J 2018 14;111(3):710. PMID:30376228

36. King CJ, Bolton A, Guerrasio J, Trosterman A. Defining Medical Student Patient Care Responsibilities Before Intern Year: Results of a National Survey. South Med J 2017;110(12):765–769. PMID:29197310

37. Edwards PJ, Roberts I, Clarke MJ, Diguiseppi C, Wentz R, Kwan I, Cooper R, Felix LM, Pratap S. Methods to increase response to postal and electronic questionnaires. Cochrane Database Syst Rev 2009 Jul 8;(3):MR000008. PMID:19588449

For peer review only

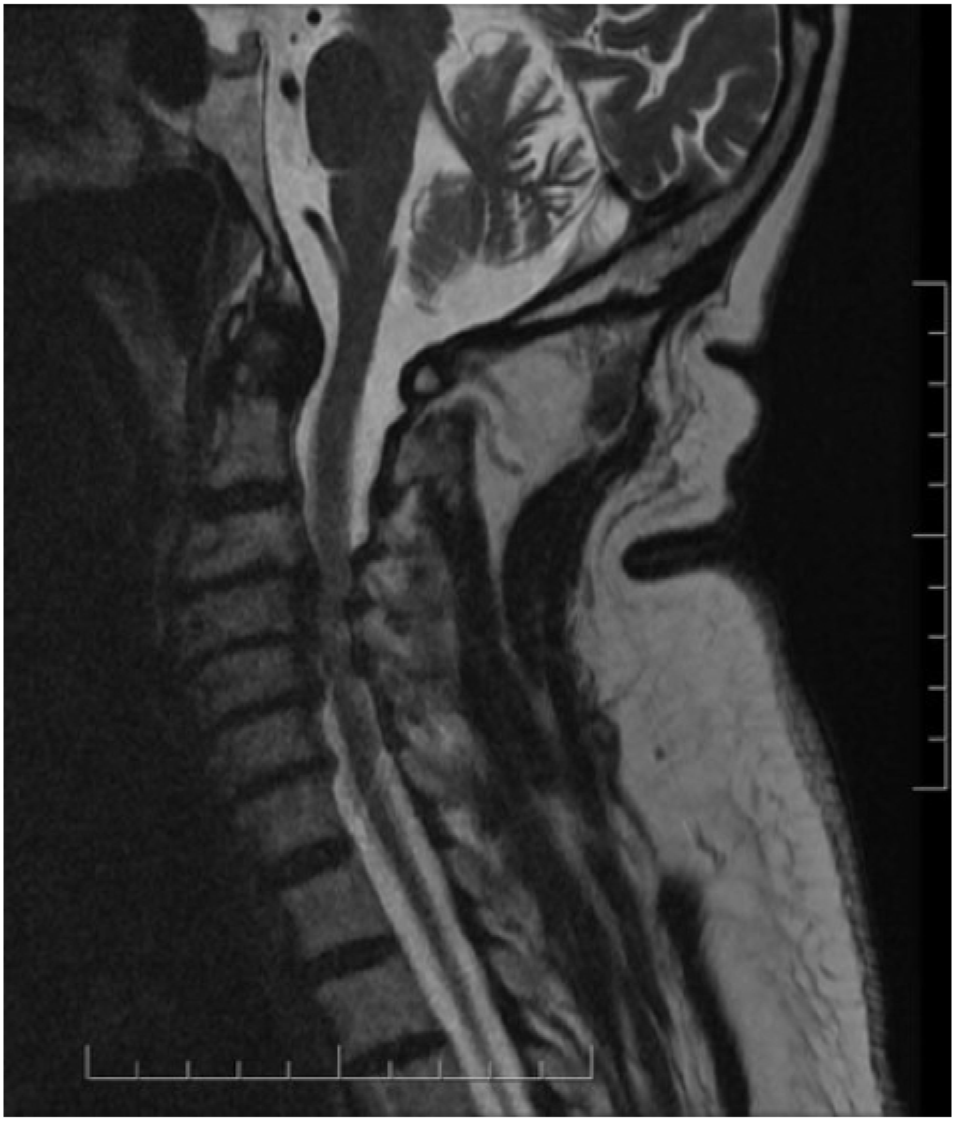

Figure 1. Typical MRI scan from patient with DCM showing spinal cord compression and multi-level degenerative changes.

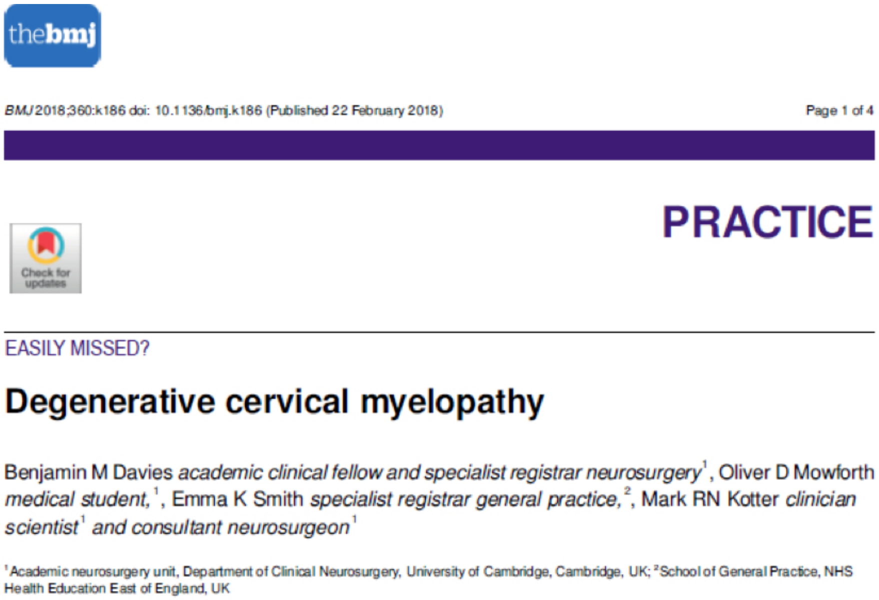

BMJ 2018;360:k186 doi: 10.1136/bmj.k186 (Published 22 February 2018) Page 1 of 4

PRACTICE

EASILY MISSED?

Degenerative cervical myelopathy

Benjamin M Davies *academic clinical fellow and specialist registrar neurosurgery*[1], Oliver D Mowforth *medical student,*[1], Emma K Smith *specialist registrar general practice,*[2], Mark RN Kotter *clinician scientist*[1] *and consultant neurosurgeon*[1]

[1]Academic neurosurgery unit, Department of Clinical Neurosurgery, University of Cambridge, Cambridge, UK; [2]School of General Practice, NHS Health Education East of England, UK

Figure 2. DCM Educational Article commissioned by the British Medical Journal.

MYELOPATHY.ORG STUDENT SOCIETY

Myelopathy Student Survey

First Question. Consent: I have read the participant information sheet and consent to my survey response being used in this study (yes/no).

Survey Part 1: Teaching

- Which medical school do you study at?
  (dropdown selection of all UK medical schools, including other category)
- Preclinical or clinical?
  (dropdown selection of preclinical or clinical)
- Which year of medical school?
  (dropdown selection: year1; year2; year3; year4; year5; year6; other)
- What is your planned/most likely career speciality?
  (free entry)
- Have you heard of degenerative cervical myelopathy (also known as cervical spondylotic myelopathy)?
  (dropdown selection: yes; no)
- Have you had any teaching on cervical myelopathy from your medical school?
  (dropdown selection: yes; no)
- What is the total duration of your cervical myelopathy teaching?
  (dropdown selection: 0 mins; 15 mins; 30 mins; 1 hour; 2 hours; >2 hours)
- What form did the teaching take?
  (dropdown selection: lecture; seminar; ward-based teaching; e-learning; other; N/A)
- How much private study have you done on cervical myelopathy?
  (dropdown selection: 0 mins; 15 minutes; 30 minutes; 1 hour; 2 hours; >2 hours)

Survey Part 2: Knowledge

For many of the following questions there is often not necessarily a right or wrong and we are often more interested in your perception.

- How would you rate your overall knowledge of cervical myelopathy?
  (dropdown selection: excellent, very good, average, poor, very poor)
- What do you estimate is the prevalence of cervical myelopathy is in the over 40s?
  (dropdown selection: 0.01%; 0.1%; 1%; 5%; 10%; 50%)
- Which do you think is the best imaging modality to diagnose myelopathy? (4 or 5 choices)
  (dropdown selection: MRI; CT; X-ray; myelography; ultrasound)
- What do you think the average time to diagnosis is from first presentation? (5 choices)
  (dropdown selection: 1 week; 1 month; 1 year; 2 years; 5 years)
- On average, how many consultations do you think there are before a patient is diagnosed?
  (dropdown selection 1;2;5;10;20)
- Do you think time to diagnosis is likely important for long-term prognosis?
  (dropdown selection: yes; no)
- What effect do you think surgery is likely to have on a patient's symptoms?
  (dropdown selection: improve; stabilise; worsen)
- Which of these symptoms might be present in cervical myelopathy?
  (clumsy gait; neck pain/stiffness; falls; imbalance; loss of bowel control; loss of urinary control; erectile dysfunction; paraesthesia; limb weakness; limb numbness; limb pain; loss of dexterity)
- Please rank these conditions from worst to best in terms of your estimate of patient quality of life?
  (dropdown selection: cancer; cervical myelopathy; diabetes; depression; heart failure)
- How would you most like to learn about cervical myelopathy in the future?
  (dropdown selection: lecture; seminar; private study; online e-learning material; not interested)

Figure 3. Final Survey Design

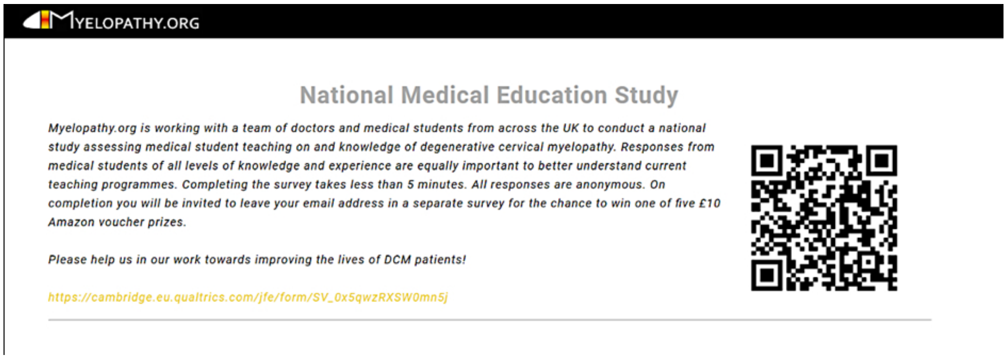

Figure 4. A standard advertisement devised by the protocol development team and approved by the research ethics committee is being used for all survey advertising.

UNIVERSITY OF CAMBRIDGE

UNIVERSITY OF CAMBRIDGE MYELOPATHY.ORG MYELOPATHY.ORG STUDENT SOCIETY

Degenerative Cervical Myelopathy (DCM): Medical Student Survey

This survey forms part of a national project assessing medical student teaching on and perception of degenerative cervical myelopathy (DCM). Do not worry if you know little or nothing about this condition. Many of the questions test your perceptions. Response from participants of all knowledge levels are equally important to our study design.

If you are currently a medical student in the UK, you are eligible to take part. Taking part is completely voluntary and confidential. All data will be identified only by a code, accessed only by the immediate research team and no personal details will be stored.

Taking part involves completing a short survey, which should take less than 5 minutes. All responses are anonymous. On completion, you will be invited to leave your email address in a separate survey to be entered into the draw to win one of five £10 Amazon voucher prizes.

In taking part you will enable us to better understand current teaching on and medical student perception of DCM. We aim to improve medical school curricula to improve outcomes for DCM patients.

We plan to publish the results of this study in a research article. You will also be able to keep updated on our study through our Myelopathy.org blog articles.

The study is being run from University of Cambridge and has received ethical approval from the Psychology Ethics Committee of the University of Cambridge.

The first question of the survey will ask you to confirm your consent to participate in the study.

Please contact us with any questions you may have: om283@cam.ac.uk

Do you consent to participate in this study? If no, please stop filling out this form.

- Yes
- No

Figure 5. Survey Front Page including Patient Information Sheet and Capturing of Informed Consent.

BMJ Open

Current Provision of Myelopathy Education in Medical Schools in the United Kingdom: Protocol for a National Medical Student Survey

Journal:	*BMJ Open*
Manuscript ID	bmjopen-2019-035563.R2
Article Type:	Protocol
Date Submitted by the Author:	20-Apr-2020
Complete List of Authors:	Mowforth, Oliver; University of Cambridge, Division of Neurosurgery, Department of Clinical Neurosciences<br>Davies, Benjamin; University of Cambridge, Division of Neurosurgery, Department of Clinical Neurosciences<br>Stewart, Max; University of Cambridge, School of Clinical Medicine<br>Smith, Sam; University of Cambridge, School of Clinical Medicine<br>Willison, Alice; University of Dundee, Ninewells Hospital and Medical School<br>Ahmed, Shahzaib; University of Cambridge, School of Clinical Medicine<br>Starkey, Michelle; Myelopathy.org<br>Sadler, Iwan; Myelopathy.org<br>Sarewitz, Ellen; Myelopathy.org<br>Stacpoole, Sybil; University of Cambridge, Neurology Unit, Department of Clinical Neurosciences<br>Kotter, Mark; University of Cambridge, Department of Clinical Neurosurgery; Ann McLaren Laboratory of Regenerative Medicine, Department of Clinical Neurosciences
**Primary Subject Heading**:	Neurology
Secondary Subject Heading:	Surgery
Keywords:	NEUROSURGERY, Neurosurgery < SURGERY, MEDICAL EDUCATION & TRAINING, NEUROLOGY

SCHOLARONE™
Manuscripts

I, the Submitting Author has the right to grant and does grant on behalf of all authors of the Work (as defined in the below author licence), an exclusive licence and/or a non-exclusive licence for contributions from authors who are: i) UK Crown employees; ii) where BMJ has agreed a CC-BY licence shall apply, and/or iii) in accordance with the terms applicable for US Federal Government officers or employees acting as part of their official duties; on a worldwide, perpetual, irrevocable, royalty-free basis to BMJ Publishing Group Ltd ("BMJ") its licensees and where the relevant Journal is co-owned by BMJ to the co-owners of the Journal, to publish the Work in this journal and any other BMJ products and to exploit all rights, as set out in our licence.

The Submitting Author accepts and understands that any supply made under these terms is made by BMJ to the Submitting Author unless you are acting as an employee on behalf of your employer or a postgraduate student of an affiliated institution which is paying any applicable article publishing charge ("APC") for Open Access articles. Where the Submitting Author wishes to make the Work available on an Open Access basis (and intends to pay the relevant APC), the terms of reuse of such Open Access shall be governed by a Creative Commons licence – details of these licences and which Creative Commons licence will apply to this Work are set out in our licence referred to above.

Other than as permitted in any relevant BMJ Author's Self Archiving Policies, I confirm this Work has not been accepted for publication elsewhere, is not being considered for publication elsewhere and does not duplicate material already published. I confirm all authors consent to publication of this Work and authorise the granting of this licence.

For peer review only

Current Provision of Myelopathy Education in Medical Schools in the United Kingdom: Protocol for a National Medical Student Survey

Oliver D. Mowforth B.A. M.B. B.Chir,[1] Benjamin M. Davies B.Sc. MRCS,[1] Max Stewart B.A,[2] Sam Smith B.A.,[2] Alice Willison B.Sc. MRes MBChB,[3], Shahzaib Ahmed B.A.,[2] Michelle L. Starkey Ph.D.,[4] Iwan Sadler,[4] Ellen Sarewitz M.A.,[4] Sybil R. Stacpoole Ph.D.,[5], Mark R. Kotter M.D. M.Phil Ph.D.[1,6]

1. Division of Neurosurgery, Department of Clinical Neurosciences, University of Cambridge, Cambridge, UK
2. School of Clinical Medicine, University of Cambridge, Cambridge, UK
3. Ninewells Hospital and Medical School, University of Dundee, Dundee, UK
4. Myelopathy.org, Cambridge, UK
5. Neurology Unit, Department of Clinical Neurosciences, University of Cambridge, Cambridge, UK
6. Anne McLaren Laboratory for Regenerative Medicine, Wellcome Trust-Medical Research Council Cambridge Stem Cell Institute, University of Cambridge, Cambridge, UK.

Key words: spinal cord diseases; cervical vertebrae; spondylosis; spinal osteophytosis; surveys and questionnaires; students, medical; education, medical

Corresponding author:

Dr Mark Kotter
Division of Neurosurgery
Department of Clinical Neurosciences
University of Cambridge
CB2 0SZ
Email: mrk25@cam.ac.uk
Telephone: +44 1223 763366
Fax: +44 1223 763350

Reference count: 35

Full text word count: 2550

Figure count: 5

For peer review only

Abstract

Introduction

Degenerative cervical myelopathy (DCM) is a common, disabling and progressive neurological condition triggered by chronic compression of the cervical spinal cord by surrounding degenerative changes. Early diagnosis and specialist management are essential to reduce disability, yet time to diagnosis is typically prolonged. Lack of sufficient representation of DCM in undergraduate and postgraduate medical curricula may contribute to the poor recognition of DCM by non-specialist doctors in clinical practice.

In this study our objective therefore is to assess DCM teaching provision in medical schools throughout the United Kingdom (UK) and to assess the impact of teaching on the DCM knowledge of UK medical students.

Methods and analysis

A 19-item questionnaire capturing data on medical student demographics, myelopathy teaching and myelopathy knowledge was designed. Ethical approval was granted by the Psychology Research Ethics Committee, University of Cambridge. An online survey was hosted on Myelopathy.org, an international myelopathy charity. Students studying at a UK medical school are eligible for inclusion. The survey is advertised nationally through university social media pages, university email bulletins and the national student network of Myelopathy.org. Advertisements are scheduled monthly over a 12-month recruitment period. Participation is incentivised by entering consenting participants of completed surveys to an Amazon voucher prize draw. Responses are anonymised using participant-chosen unique identifier codes. A participant information sheet followed by an explicit survey question captured participant informed consent. The study is due to report in Summer 2020. Regular updates on the progress of the study will be published on Myelopathy.org.

Ethics and Dissemination

For peer review only

Ethical Approval for the study was granted by the Psychology Research Ethics Committee, University of Cambridge (PRE.2018.099). Insurance cover was arranged via the University of Cambridge (HVS/2018/2366). The findings of the study described in this protocol, and all other related work, will be submitted for publication in a peer-reviewed journal and will be presented at scientific conferences.

For peer review only

Strengths and limitations of this study

- This is a large national questionnaire-based study targeted at all medical students in the United Kingdom
- This is the first study to assess DCM medical education in the UK
- This is a student-led study with oversight from senior researchers and involvement of DCM patients
- The study data will provide insight into an area that has potential to translate into substantial improvements in patient outcomes
- Risk of selection bias may overestimate the current knowledge of DCM among UK medical students

For peer review only

Introduction

Degenerative cervical myelopathy (DCM) is a common neurological condition of symptomatic cervical spinal cord compression due to degenerative changes of the spine (Figure 1).[1] DCM is the most common cause of spinal cord impairment in adults worldwide, with an estimated prevalence of up to 5% in the over 40s.[1],[2],[3].

Substantial improvements in the care of DCM patients could be realised today without any additional scientific understanding of the condition.[1] DCM patients almost universally experience diagnostic delays. For example, one study demonstrated that on average it took 2 years and 5 clinic appointments before the diagnosis was made.[4] Improvement in diagnosis is therefore a key target which would provide immediate reductions in disability.[5]

Initially, DCM patients are commonly seen by primary care, emergency and medical physicians.[6],[7] These non-specialist triage points appear key to earlier diagnosis, yet DCM features poorly in medical curricula.[8] This risks non-specialist doctors being poorly equipped to detect DCM.

The current focus and makeup of DCM medical education is unclear. Our recent evaluation of undergraduate and post-graduate medical curricula identified deficiencies in DCM representation, however collectively this did not correlate with student performance in multiple-choice question banks.[8]

The aim of this study is to describe the teaching of DCM in UK medical schools and to assess the influence on medical student knowledge of DCM. Ultimately, the aim is to identify both training and knowledge gaps to inform guidance on DCM education in order to address the burden and impact of delayed diagnosis. We hypothesise that knowledge and awareness amongst medical professionals is related to their specific medical education on DCM.

For peer review only

Methods and analysis

Study Partners

Myelopathy.org

Myelopathy.org is an international cervical myelopathy charity, which aims to improve health and well-being in DCM.[9] This study forms part of a wider project to evaluate DCM education and awareness amongst medical professionals in the UK, and develop focused and effective interventions to improve this. This includes our recent gap-analysis of key medical resources and knowledge at each stage of the primary care training pathway[10] and an educational article on DCM for non-specialist medical professionals, commissioned by the British Medical Journal (Figure 2).[1] The charity is focussed on understanding how medical education can be improved as one possible strategy to address the current problems of delayed, missed and under-diagnosis.[1]

Student Society of Myelopathy.org

The foundations of professional knowledge begin with students' formative experiences at medical school. Therefore, the Student Society of Myelopathy.org was established in 2018 with the aim of improving DCM engagement and awareness amongst medical students. The society remains in its infancy but has hosted a series of educational talks at the University of Cambridge; established national medical student essay and research prizes; designed and initiated this current study; and secured sponsorship from corporate partners to support these projects.

Study Design

An electronic survey was agreed to be the most efficient way to simultaneously assess teaching, perceptions of teaching, and current knowledge, and to efficiently reach students from all UK medical schools. Part One of the survey was designed to capture data on basic demographic characteristics and teaching of DCM in UK medical schools. Part Two was designed to capture data on knowledge and awareness of fundamental DCM facts including prevalence, symptoms, diagnostic imaging modalities and surgical effectiveness in addition

For peer review only

to appreciation of important current challenges such as prolonged time to diagnosis and poor patient quality of life. Part Two was designed to capture participant perception of their DCM knowledge. These questions were considered to be a reasonable balance of capturing sufficient data whilst maintaining an acceptably short survey completion time of approximately 5 minutes.

The study management team comprised representation from second year to final year students from multiple UK medical schools. The team comprised an academic neurosurgical registrar [BMD] and an academic neurosurgical consultant [MRK], both with special clinical and academic interests in DCM, and an academic consultant neurologist [SRS] who is a neuroscience teaching fellow and neuroscience course organiser at the University of Cambridge. Importantly, this is a student-motivated, student-led project with supervision, oversight and mentorship at all stages of the study from a team of senior researchers.

The survey was iteratively refined amongst the study management group until section one was felt to capture sufficient information on current DCM teaching provision in UK medical schools. Similarly, section two was refined until it was felt to evaluate fundamental DCM facts at a level sufficient for a non-specialist, but qualified doctor to appropriately suspect, investigate, diagnose and re-direct care in DCM. In total, a 19-item questionnaire was developed (Figure 3).

Survey development closely aligns with the AMEE 7 step guide to medical education survey development: (1) literature review; (2) focus group establishment (3) synthesis of literature review/focus group (4) item development (5) expert validation (6) feedback and expert validation and (7) piloting, [11] with the exception that a quantitative gap analysis was performed in the place of a literature review due to the lack of research on DCM medical education at the time of study design.[8] Nonetheless, planning for a systematic review of similar medical education surveys in other diseases is underway.

Survey Piloting

The survey was piloted in January 2019 by a group of 20 medical students at the University of Cambridge. These students were not directly involved in the design of the study. Data were inspected to identify any inconsistencies, ambiguities and problematic survey items. No issues were identified. Students in the pilot group were contacted to ask if they had encountered any difficulties, areas of uncertainty or had suggestions for improving the survey Students reported the survey to be acceptable on both desktop and mobile devices. Informal

For peer review only - http://bmjopen.bmj.com/site/about/guidelines.xhtml

cognitive interviews of 5 students revealed that survey items were interpreted by students as intended by the study team, without ambiguity. No further alterations were therefore made. No formal statistical data analysis was conducted at the pilot stage.

Survey Administration

To maximise reach across the UK, the survey was hosted on Qualtrics Survey Software (Provo, United States), a professional online survey platform. Qualtrics Survey Software includes both desktop and mobile-compatible versions, maximising convenience for participants.

The survey comprises two pages. The first page acts as a front page consisting of a participant information sheet and a question capturing informed consent. The second page is the main survey page. It comprises 19 questions over a single page to minimise the numbers of clicks required for participants to complete the survey.

Survey Dissemination

A network of Myelopathy.org Student Representatives was recruited, with representation of students from 25 different medical schools across the UK. Some but not all were also representatives for the Society of British Neurological Surgeons (SBNS) Neurology and Neurosurgery Student Interest Group (NANSIG). A standard advertisement devised by the protocol development team and approved by the research ethics committee is utilised for all advertising (Figure 4). Representatives from all medical schools are sent monthly reminder emails to disseminate adverts at their institution over a 12-month period from February 2019 – January 2020. There was no difference in the timing of reminder emails between different medical schools. Although prompted to advertise monthly, the final decision on frequency of advertising at each institution is left to the discretion of representatives, guided by local rules.

On completion of the survey participants are invited to leave an email address in a separate Google Forms survey to be entered into a prize draw to win one of five £10 Amazon vouchers. The prize draw was agreed by the study management group and approved by the research ethics committee. The rationale was that prizes of this value may encourage participation without providing an inappropriate incentive to participate.

The survey is advertised in social media groups and on social media pages for relevant student groups and societies at universities across the UK. For example, Facebook groups of surgical, neurosurgical, neurological and other medical student societies were approached. In

For peer review only

addition, university mailing lists and email bulletins are being utilised, where appropriate, in accordance with local rules. Adverts are also placed in the monthly NANSIG newsletter, emailed out to medical student subscribers across the UK. The survey link is also hosted on the Student Society of Myelopathy.org webpage and Facebook page.

Eligibility and Representation

All UK medical students are eligible to complete the survey. We aim to secure representation of all UK medical schools.

Consent and Confidentiality

A patient information sheet (PIS) including a question capturing informed consent forms the survey frontpage (Figure 5). The PIS emphasises the rational, purpose, study aims and the voluntariness of participation. By necessity the background information on DCM in the PIS is minimal to avoid compromising the DCM knowledge-based questions in the survey. A page block is utilised to separate the front and main survey pages.

Participants assign themselves a unique identifier based on a defined combination of characters from their mother's maiden name, the street they grew up on and their mobile phone number; this ensures anonymity and confidentiality whilst allowing the possibility of linking together future surveys from the same participant.

Data Security

All data are stored exclusively on the secure, online Qualtrics platform until closure of the survey. Thereafter, survey data will be extracted directly from the Qualtrics platform into a password-encrypted Excel spreadsheet (Microsoft, California) on a password-protected computer. Only the immediate research team will be granted access to this on a need-to-access basis. After completion of data analysis, all data that is no longer required will be deleted. No participant-identifiable data will be collected or stored at any point.

Statistical Analysis

Data will be analysed in Microsoft Excel and using statistical software.

Relationships between duration of DCM teaching, duration of private study on DCM and student perception of their DCM knowledge and objective performance in the DCM knowledge-based questions will be assessed. Differences in performance on the knowledge-based questions will be compared between groups based on medical school, year of medical

For peer review only

school, duration of teaching, format of teaching, duration of private study, prior self-reported awareness of DCM, self-rating of DCM knowledge and between groups of medical students wishing to pursue neurological/neurosurgical career specialties and those who do not.

Due to all items in the survey being mandatory, we anticipate that missing data will be minimal, however will conduct a missing data analysis as necessary. It is not expected the data will be suitable for imputation, thus we will compare demographics to establish any selection bias caused by the exclusion of missing values. We will perform analysis on all collected values. The survey findings will be presented using descriptive statistics. Inferential statistical tests may be used to consider the relationship between knowledge and educational provision. Distribution testing will be performed. Appropriate statistical methods will be selected based on the class and distribution of the data; it is anticipated data will be non-parametric, and as measures are recorded on an ordinal or categorial basis, will therefore be compared using tests such as Chi-Squared or Kruksall Wallis Tests.

Patient and Public Involvement

Patients were involved in the conception, design, development and conduct of this study. A DCM Patient and Public Engagement day hosted at the University of Cambridge was captured by Cambridge TV in a documentary.[12] During this day, a focus group of DCM patients emphasised their experiences of diagnostic delays. This has been frequently echoed by patients in Myelopathy.org social media groups and in a DCM word-cloud creating initiative to understand patient’s perceptions of the condition for a related project. DCM patients therefore inspired the conception of this study and approved the final survey design. Patients also assisted in the essential administrative tasks of the project, such as logo design. The online survey for the study was hosted on Myelopathy.org, which is a patient-maintained website for people with DCM. Patients were, therefore, active in aiding the dissemination of the survey to UK medical students. Patients who were involved in preparation of the manuscript are among the authors. DCM patients are involved in plans to disseminate this research to the patient community, including blog articles on Myelopathy.org, posts in online patient support groups and presence at spinal conferences in the UK.

For peer review only - http://bmjopen.bmj.com/site/about/guidelines.xhtml

Discussion

Study Rationale

This is the first study to describe the DCM education at medical schools nationally. Medical students are recruited to an online survey, including an assessment of their DCM knowledge and their personal DCM learning experience. By comparison of learning experience and knowledge, and also comparison between medical schools, it is anticipated both the presence or absence of a knowledge gap can be evaluated, and the more successful teaching formats identified. This will be used to inform Myelopathy.org education recommendations.

Why assess DCM education?

DCM is a devastating condition with one of the poorest quality of life scores of any chronic disease.[13] Symptoms include loss of manual dexterity, gait impairment, limb paraesthesia, limb weakness, limb stiffness, bladder and bowel disturbance, neck pain and neck stiffness.[1],[14],[15] The full spectrum of symptoms and the natural history of DCM is poorly appreciated,[16], [17] yet it remains clear that the condition poses a substantial burden on the physical and mental health of people with DCM [18] and their supporters [19]. There is a broad differential for DCM symptoms[20] and poor consensus on what the most common DCM symptoms are. In addition, an increasing number of atypical symptoms, such as dysaesthetic sensory symptoms,[6] chest tightness,[21] and headache[22] are being reported.[23] Neurological examination findings include upper motor neuron signs in the upper and lower limbs: spasticity and hyperreflexia in addition to weakness.[1],[15].

The latest international guidelines[24] recommend surgical decompression as the only evidence-based management [25],[26] for patients with moderate to severe, or progressive DCM. Surgery is also a management option in patients with mild DCM.[24] Importantly, surgery can halt progression of the condition but is ineffective at reversing existing damage.[26] Therefore, the traditional medical dogma that prevention is better than cure holds as strongly as ever in DCM: time is spine.[1]

Scientific advances are sorely needed to improve therapeutic options and ultimately to cure disabled patients with DCM.[27], [28] However, whilst research into the neurobiology of DCM and neural regeneration,[29] such as the RECEDE-MYELOPATHY and CSM-PROTECT trials offer hope for the future, [9],[30],[31] even the most optimistic scientist

For peer review only

would concede that tangible benefits for patients arising from these technologies remain on the horizon: one systematic review reported average duration of knowledge translation of 17 years from research to practice.[32]

Therefore, pragmatic approaches are required to improve outcomes for today's DCM patients. One such approach entails addressing the substantial problem of diagnostic delays[1], which is expected to improve outcomes in this progressive condition.[5] One method of instituting this may be through better education. Indeed, a phenomenon of "neurophobia" is well-described amongst medical students and non-specialist doctors.[33],[34],[35] Additionally, evidence from our recent gap analysis suggests poor representation of DCM in medical curricula.[10] These factors may point to a knowledge gap in DCM medical education.

Benefits of a medical student survey

Assessing teaching and knowledge among medical students is a pragmatic and initial step to evaluate a knowledge gap. Medical school is the foundation of all medical professionals' education and is especially important for instilling basic knowledge of important areas of medicine that may fall outside a doctor's future field of specialisation. It is likely that this is particularly pertinent for knowledge that this not later core (i.e. built on or refined) during specialisation. For this purpose, a survey of medical students is best placed to simultaneously assess knowledge, whilst reporting on the current provision of DCM teaching and perceptions of it. Moreover, the survey itself may help to prime an audience to support a subsequent intervention, or at the very least raise awareness.

Urgent improvements in outcomes for DCM patients are needed. Earlier diagnosis would improve outcomes based on current treatments. This study aims to evaluate DCM education and knowledge, and explore their relationship, amongst medical students. It is hoped this will inform educational interventions that may offer the greatest chance of shaping the non-specialist doctor's diagnosis of DCM tomorrow.

Limitations

Online surveys are an effective method of reaching students from diverse medical schools.[36] In addition, digital collection of data offers a secure and seamless stream, facilitating data analysis. However, their limitation is in understanding how representative the findings are.

For peer review only

In order to mitigate this, a number of different dissemination approaches are being employed. This includes the appointment of local representatives at each UK medical school, the use social media posts, society mailing lists; society newsletters; medical school email bulletins; medical school newsletters, and also the use of prizes as an incentive.[37] Additionally, the capture of participant demographics will help to measure the sample characteristics.

The engagement with open surveys, often involves some predefined interest. It is possible therefore a selection-bias towards students interested in neuroscience is more likely and results would be considered as a best-case scenario. Whilst there is a risk of overlooking or underestimating the magnitude of a knowledge gap given the outlined benefits of this methodology, this is an accepted risk. Whatever the relationship between DCM teaching and knowledge turns out to be, it will be instructive with regards to the overall objective of identifying ways of improving DCM diagnosis.

We acknowledge that wording of the survey item "Do you think time to diagnosis is important for long term prognosis" could be perceived a leading and would have been better phrased in a more neutral format such as "How important is time to diagnosis for long-term prognosis?". We will monitor the performance of this item closely in our analysis.

Ethics and Dissemination

Ethical Approval for the study was granted by the Psychology Research Ethics Committee, University of Cambridge (PRE.2018.099). Insurance cover was arranged via the University of Cambridge (HVS/2018/2366). The findings of the study described in this protocol, and all other related work, will be submitted for publication in a peer-reviewed journal and will be presented at scientific conferences.

For peer review only

Author contributions:

ODM, BMD, IS, ES, SRS and MRK were involved in the conceptualisation and design of this study. ODM wrote the first draft of the manuscript. ODM, BMD, MS, SS, AW, SA, MLS, IS, ES, SRS and MRK reviewed and approved the final manuscript.

Funding:

Research in the MRNK's laboratory is supported by a core support grant from the Wellcome Trust and MRC to the Wellcome Trust-Medical Research Council Cambridge Stem Cell Institute. MRNK is supported by a NIHR Clinician Scientist Award and BMD is supported by a NIHR Clinical Doctoral Research Fellowship.

Disclaimer:

This report is independent research arising from a Clinician Scientist Award, CS-2015-15-023, supported by the National Institute for Health Research. The views expressed in this publication are those of the authors and not necessarily those of the NHS, the National Institute for Health Research or the Department of Health and Social Care.

Presented at:

World Federation of Neurosurgical Societies International Meeting, Belgrade, Serbia, March 2019.

British Neurosurgical Research Group Meeting, Edinburgh, November 2019

Competing Interests:

ODM, BMD, MS, SS, AW, SA, MLS, IS, ES and MRK have voluntary roles at Myelopathy.org, an international DCM charity.

Patient consent for publication: not required

Ethical approval: approved by Psychology Research Ethics Committee, University of Cambridge

Data availability statement: All data relevant to the study are included in the article or uploaded as online supplementary information.

For peer review only

Acknowledgements:

The authors are grateful to all the patients who have inspired this study, most especially at the University of Cambridge Patient and Public Engagement Day. In addition, the authors would like to thank the Student Society of Myelopathy.org and NANSIG medical student representatives for their assistance in disseminating the research survey.

For peer review only

Figure Legends:

Figure 1. Typical MRI scan from patient with DCM showing spinal cord compression and multi-level degenerative changes.

Figure 2. DCM Educational Article commissioned by the British Medical Journal. Reproduced with permission from British Medical Journal.

Figure 2. Final Survey Design. Reproduced with permission from Myelopathy.org.

Figure 4. A standard advertisement devised by the protocol development team and approved by the research ethics committee is being used for all survey advertising. Reproduced with permission from Myelopathy.org.

Figure 5. Survey Front Page including Patient Information Sheet and Capturing of Informed Consent. Reproduced with permission from Myelopathy.org.

For peer review only

References

1. Davies BM, Mowforth OD, Smith EK, Kotter MR. Degenerative cervical myelopathy. BMJ 2018 Feb 22;360:k186. PMID:29472200

2. Kovalova I, Kerkovsky M, Kadanka Z, Kadanka Z, Nemec M, Jurova B, Dusek L, Jarkovsky J, Bednarik J. Prevalence and Imaging Characteristics of Nonmyelopathic and Myelopathic Spondylotic Cervical Cord Compression: SPINE 2016 Dec;41(24):1908–1916. [doi: 10.1097/BRS.0000000000001842]

3. Nouri A, Tetreault L, Singh A, Karadimas SK, Fehlings MG. Degenerative Cervical Myelopathy: Epidemiology, Genetics, and Pathogenesis. Spine 2015 Jun 15;40(12):E675-693. PMID:25839387

4. Behrbalk E, Salame K, Regev GJ, Keynan O, Boszczyk B, Lidar Z. Delayed diagnosis of cervical spondylotic myelopathy by primary care physicians. Neurosurg Focus 2013 Jul;35(1):E1. PMID:23815245

5. Cates JR, Soriano MM. Cervical spondylotic myelopathy. J Manipulative Physiol Ther 1995 Sep;18(7):471–475. PMID:8568430

6. Mowforth OD, Davies BM, Kotter MR. “I am not delusional!” Sensory dysaesthesia secondary to degenerative cervical myelopathy. BMJ Case Rep 2019 Apr 11;12(4). PMID:30975783

7. Radcliff KE, Curry EP, Trimba R, Walker JB, Purtill JJ, Austin MS, Parvizi J, Vaccaro AR, Hilibrand AS, Albert TJ. High Incidence of Undiagnosed Cervical Myelopathy in Patients With Hip Fracture Compared With Controls. J Orthop Trauma 2016 Apr;30(4):189–193. PMID:26562581

8. Waqar M, Wilcock J, Garner J, Davies B, Kotter M. Quantitative analysis of medical students’ and physicians’ knowledge of degenerative cervical myelopathy. BMJ Open 2020 Jan 12;10(1):e028455. PMID:31932384

9. Neurology TL. A focus on patient outcomes in cervical myelopathy. Lancet Neurol 2019 Jul 1;18(7):615. PMID:31202463

10. Davies B, Wilcock J, Garner J, Waqar M, Kotter M. A quantitative analysis of medical students’ and physicians’ knowledge of degenerative cervical myelopathy. 2019 Jun 10 [cited 2019 Sep 15]; [doi: 10.17863/CAM.40662]

11. Developing questionnaires for educational research: AMEE Guide No. 87: Medical Teacher: Vol 36, No 6 [Internet]. [cited 2020 Mar 13]. Available from: https://www.tandfonline.com/doi/full/10.3109/0142159X.2014.889814

12. Roberts E. Elemental Ideas: Cervical Spondylotic Myelopathy. http://www.myelopathy.org/the-csm-documentary.html.

For peer review only

13. Oh T, Lafage R, Lafage V, Protopsaltis T, Challier V, Shaffrey C, Kim HJ, Arnold P, Chapman J, Schwab F, Massicotte E, Yoon T, Bess S, Fehlings M, Smith J, Ames C. Comparing Quality of Life in Cervical Spondylotic Myelopathy with Other Chronic Debilitating Diseases Using the SF-36 Survey. World Neurosurg 2017 Jan 5; PMID:28065875

14. Arnold JG. The clinical manifestations of spondylochondrosis (spondylosis) of the cervical spine. Ann Surg 1955 Jun;141(6):872–889. PMID:14377330

15. Brain WR, Northfield D, Wilkinson M. The neurological manifestations of cervical spondylosis. Brain J Neurol 1952 Jun;75(2):187–225. PMID:14934989

16. Badhiwala JH, Wilson JR. The Natural History of Degenerative Cervical Myelopathy. Neurosurg Clin N Am 2018 Jan;29(1):21–32. PMID:29173433

17. Clair SS, Bell GR. Natural History of Cervical Spondylotic Myelopathy. Semin Spine Surg 2007 Mar 1;19(1):2–5. [doi: 10.1053/j.semss.2007.01.005]

18. Stoffman MR, Roberts MS, King JT. Cervical spondylotic myelopathy, depression, and anxiety: a cohort analysis of 89 patients. Neurosurgery 2005 Aug;57(2):307–313; discussion 307-313. PMID:16094160

19. Mowforth OD, Davies B, Kotter M. Poor quality of life in degenerative cervical myelopathy carers (Preprint). 2019 Jul 25 [cited 2019 Oct 21]; [doi: 10.17863/CAM.42010]

20. Tracy JA, Bartleson JD. Cervical spondylotic myelopathy. The Neurologist 2010 May;16(3):176–187. PMID:20445427

21. Kobayashi H, Kikuchi S, Otani K, Sekiguchi M, Sekiguchi Y, Konno S. Development of a self-administered questionnaire to screen patients for cervical myelopathy. BMC Musculoskelet Disord 2010 Nov 22;11:268. PMID:21092213

22. Schrot RJ, Mathew JS, Li Y, Beckett L, Bae HW, Kim KD. Headache relief after anterior cervical discectomy: post hoc analysis of a randomized investigational device exemption trial. J Neurosurg Spine 2014 Aug;21(2):217–222. PMID:24836655

23. Davies BM, Munro CF, Kotter MR. A Novel Insight Into the Challenges of Diagnosing Degenerative Cervical Myelopathy Using Web-Based Symptom Checkers. J Med Internet Res 2019 11;21(1):e10868. PMID:30300137

24. Fehlings MG, Tetreault LA, Riew KD, Middleton JW, Aarabi B, Arnold PM, Brodke DS, Burns AS, Carette S, Chen R, Chiba K, Dettori JR, Furlan JC, Harrop JS, Holly LT, Kalsi-Ryan S, Kotter M, Kwon BK, Martin AR, Milligan J, Nakashima H, Nagoshi N, Rhee J, Singh A, Skelly AC, Sodhi S, Wilson JR, Yee A, Wang JC. A Clinical Practice Guideline for the Management of Patients With Degenerative Cervical Myelopathy: Recommendations for Patients With Mild, Moderate, and Severe Disease and Nonmyelopathic Patients With Evidence of Cord Compression. Glob Spine J 2017 Sep 1;7(3_suppl):70S-83S. [doi: 10.1177/2192568217701914]

25. Fehlings MG, Ibrahim A, Tetreault L, Albanese V, Alvarado M, Arnold P, Barbagallo G, Bartels R, Bolger C, Defino H, Kale S, Massicotte E, Moraes O, Scerrati M, Tan G,

For peer review only

Tanaka M, Toyone T, Yukawa Y, Zhou Q, Zileli M, Kopjar B. A global perspective on the outcomes of surgical decompression in patients with cervical spondylotic myelopathy: results from the prospective multicenter AOSpine international study on 479 patients. Spine 2015 Sep 1;40(17):1322–1328. PMID:26020847

26. Fehlings MG, Wilson JR, Kopjar B, Yoon ST, Arnold PM, Massicotte EM, Vaccaro AR, Brodke DS, Shaffrey CI, Smith JS, Woodard EJ, Banco RJ, Chapman JR, Janssen ME, Bono CM, Sasso RC, Dekutoski MB, Gokaslan ZL. Efficacy and safety of surgical decompression in patients with cervical spondylotic myelopathy: results of the AOSpine North America prospective multi-center study. J Bone Joint Surg Am 2013 Sep 18;95(18):1651–1658. PMID:24048552

27. Mowforth OD, Davies BM, Goh S, O'Neill CP, Kotter MRN. Research Inefficiency in Degenerative Cervical Myelopathy: Findings of a Systematic Review on Research Activity Over the Past 20 Years. Glob Spine J 2019 Jun 12;2192568219847439. [doi: 10.1177/2192568219847439]

28. Davies BM, Khan DZ, Mowforth OD, McNair AGK, Gronlund T, Kolias AG, Tetreault L, Starkey ML, Sadler I, Sarewitz E, Houlton D, Carter J, Kalsi-Ryan S, Aarabi B, Kwon BK, Kurpad SN, Harrop J, Wilson JR, Grossman R, Curt A, Fehlings MG, Kotter MRN. RE-CODE DCM (REsearch Objectives and Common Data Elements for Degenerative Cervical Myelopathy): A Consensus Process to Improve Research Efficiency in DCM, Through Establishment of a Standardized Dataset for Clinical Research and the Definition of the Research Priorities. Glob Spine J 2019 May;9(1 Suppl):65S-76S. PMID:31157148

29. Kalsi-Ryan S, Karadimas SK, Fehlings MG. Cervical Spondylotic Myelopathy The Clinical Phenomenon and the Current Pathobiology of an Increasingly Prevalent and Devastating Disorder. The Neuroscientist 2013 Aug 1;19(4):409–421. PMID:23204243

30. Fehlings MG, Wilson JR, Karadimas SK, Arnold PM, Kopjar B. Clinical evaluation of a neuroprotective drug in patients with cervical spondylotic myelopathy undergoing surgical treatment: design and rationale for the CSM-Protect trial. Spine 2013 Oct 15;38(22 Suppl 1):S68-75. PMID:23962993

31. Kato S, Fehlings M. Degenerative cervical myelopathy. Curr Rev Musculoskelet Med 2016 Jun 1;9(3):263–271. PMID:27250040

32. Morris ZS, Wooding S, Grant J. The answer is 17 years, what is the question: understanding time lags in translational research. J R Soc Med 2011 Dec;104(12):510–520. PMID:22179294

33. Pokryszko-Dragan A, Mottershead J, Aitken G. Attitudes towards neurology among medical undergraduates. Neurol Neurochir Pol 2019;53(1):61–73. [doi: 10.5603/PJNNS.a2018.0004]

34. Shiels L, Majmundar P, Zywot A, Sobotka J, Lau CSM, Jalonen TO. Medical student attitudes and educational interventions to prevent neurophobia: a longitudinal study. BMC Med Educ 2017 Nov 21;17(1):225. PMID:29162061

For peer review only

35. Conway S, Tubridy N. “Neurophobia”: More Nurture than Nature? Ir Med J 2018 14;111(3):710. PMID:30376228

36. King CJ, Bolton A, Guerrasio J, Trosterman A. Defining Medical Student Patient Care Responsibilities Before Intern Year: Results of a National Survey. South Med J 2017;110(12):765–769. PMID:29197310

37. Edwards PJ, Roberts I, Clarke MJ, Diguiseppi C, Wentz R, Kwan I, Cooper R, Felix LM, Pratap S. Methods to increase response to postal and electronic questionnaires. Cochrane Database Syst Rev 2009 Jul 8;(3):MR000008. PMID:19588449

For peer review only

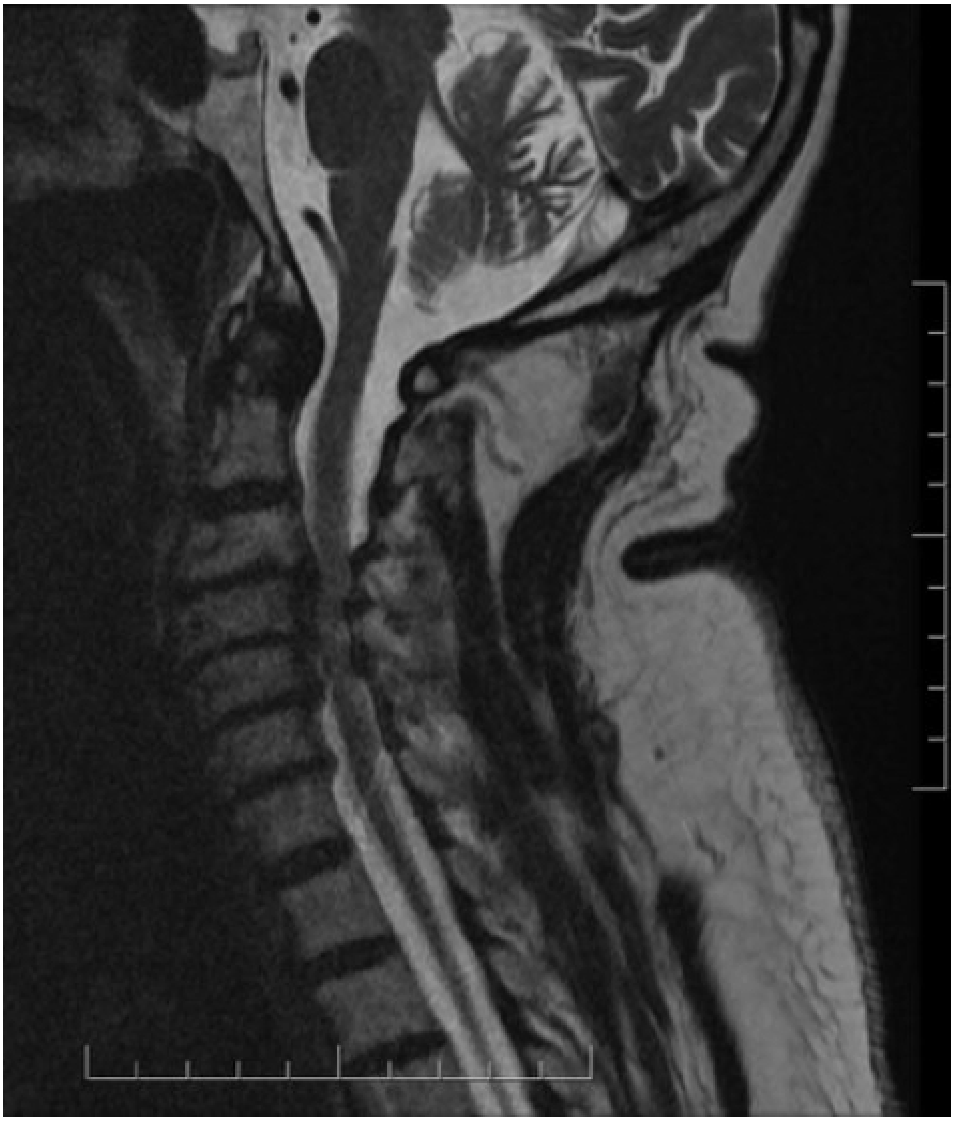

Figure 1. Typical MRI scan from patient with DCM showing spinal cord compression and multi-level degenerative changes.

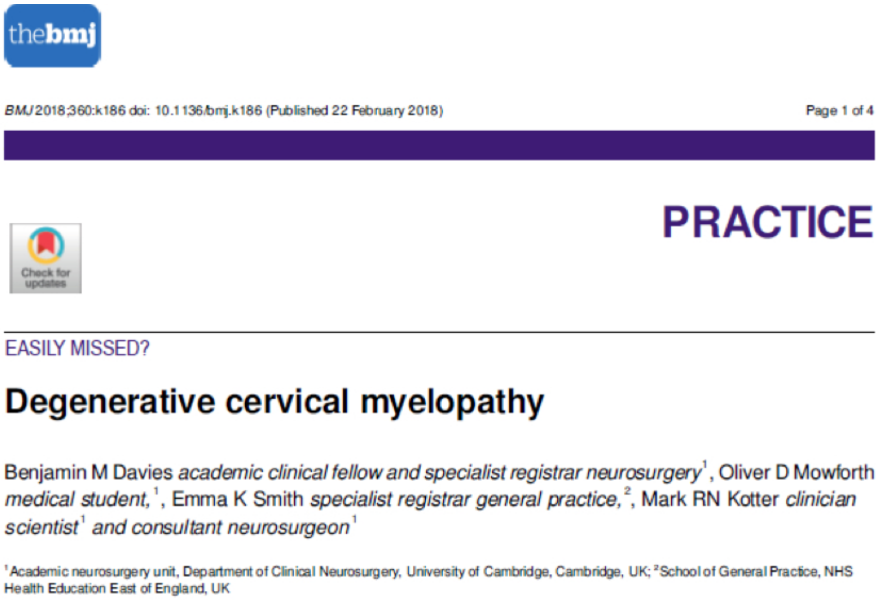
thebmj

BMJ 2018;360:k186 doi: 10.1136/bmj.k186 (Published 22 February 2018) Page 1 of 4

PRACTICE

Check for updates

EASILY MISSED?

Degenerative cervical myelopathy

Benjamin M Davies *academic clinical fellow and specialist registrar neurosurgery*[1], Oliver D Mowforth *medical student,*[1], Emma K Smith *specialist registrar general practice,*[2], Mark RN Kotter *clinician scientist*[1] *and consultant neurosurgeon*[1]

[1]Academic neurosurgery unit, Department of Clinical Neurosurgery, University of Cambridge, Cambridge, UK; [2]School of General Practice, NHS Health Education East of England, UK

Figure 2. DCM Educational Article commissioned by the British Medical Journal. Reproduced with permission from British Medical Journal.

MYELOPATHY.ORG
STUDENT SOCIETY

Myelopathy Student Survey

First Question. Consent: I have read the participant information sheet and consent to my survey response being used in this study (yes/no).

Survey Part 1: Teaching

- Which medical school do you study at?
  (dropdown selection of all UK medical schools, including other category)
- Preclinical or clinical?
  (dropdown selection of preclinical or clinical)
- Which year of medical school?
  (dropdown selection: year1; year2; year3; year4; year5; year6; other)
- What is your planned/most likely career speciality?
  (free entry)
- Have you heard of degenerative cervical myelopathy (also known as cervical spondylotic myelopathy)?
  (dropdown selection: yes; no)
- Have you had any teaching on cervical myelopathy from your medical school?
  (dropdown selection: yes; no)
- What is the total duration of your cervical myelopathy teaching?
  (dropdown selection: 0 mins; 15 mins; 30 mins; 1 hour; 2 hours; >2 hours)
- What form did the teaching take?
  (dropdown selection: lecture; seminar; ward-based teaching; e-learning; other; N/A)
- How much private study have you done on cervical myelopathy?
  (dropdown selection: 0 mins; 15 minutes; 30 minutes; 1 hour; 2 hours; >2 hours)

Survey Part 2: Knowledge

For many of the following questions there is often not necessarily a right or wrong and we are often more interested in your perception.

- How would you rate your overall knowledge of cervical myelopathy?
  (dropdown selection: excellent, very good, average, poor, very poor)
- What do you estimate is the prevalence of cervical myelopathy is in the over 40s?
  (dropdown selection: 0.01%; 0.1%; 1%; 5%; 10%; 50%)
- Which do you think is the best imaging modality to diagnose myelopathy? (4 or 5 choices)
  (dropdown selection: MRI; CT; X-ray; myelography; ultrasound)
- What do you think the average time to diagnosis is from first presentation? (5 choices)
  (dropdown selection: 1 week; 1 month; 1 year; 2 years; 5 years)
- On average, how many consultations do you think there are before a patient is diagnosed?
  (dropdown selection 1;2;5;10;20)
- Do you think time to diagnosis is likely important for long-term prognosis?
  (dropdown selection: yes; no)
- What effect do you think surgery is likely to have on a patient's symptoms?
  (dropdown selection: improve; stabilise; worsen)
- Which of these symptoms might be present in cervical myelopathy?
  (clumsy gait; neck pain/stiffness; falls; imbalance; loss of bowel control; loss of urinary control; erectile dysfunction; paraesthesia; limb weakness; limb numbness; limb pain; loss of dexterity)
- Please rank these conditions from worst to best in terms of your estimate of patient quality of life?
  (dropdown selection: cancer; cervical myelopathy; diabetes; depression; heart failure)
- How would you most like to learn about cervical myelopathy in the future?
  (dropdown selection: lecture; seminar; private study; online e-learning material; not interested)

Figure 3. Final Survey Design. Reproduced with permission from Myelopathy.org.

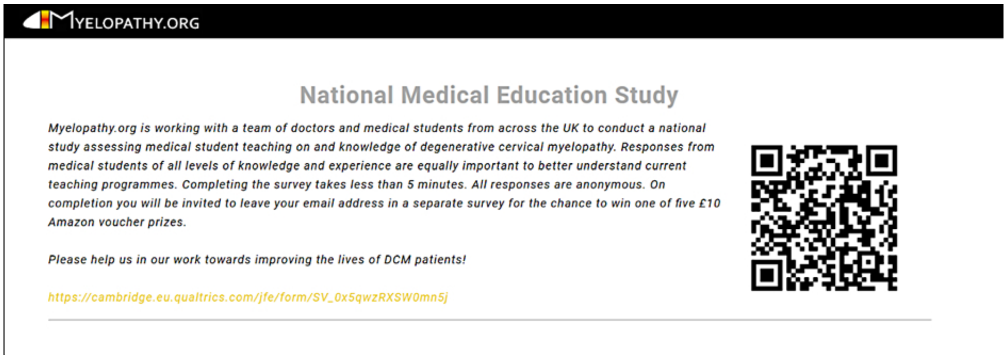

Figure 4. A standard advertisement devised by the protocol development team and approved by the research ethics committee is being used for all survey advertising. Reproduced with permission from Myelopathy.org.

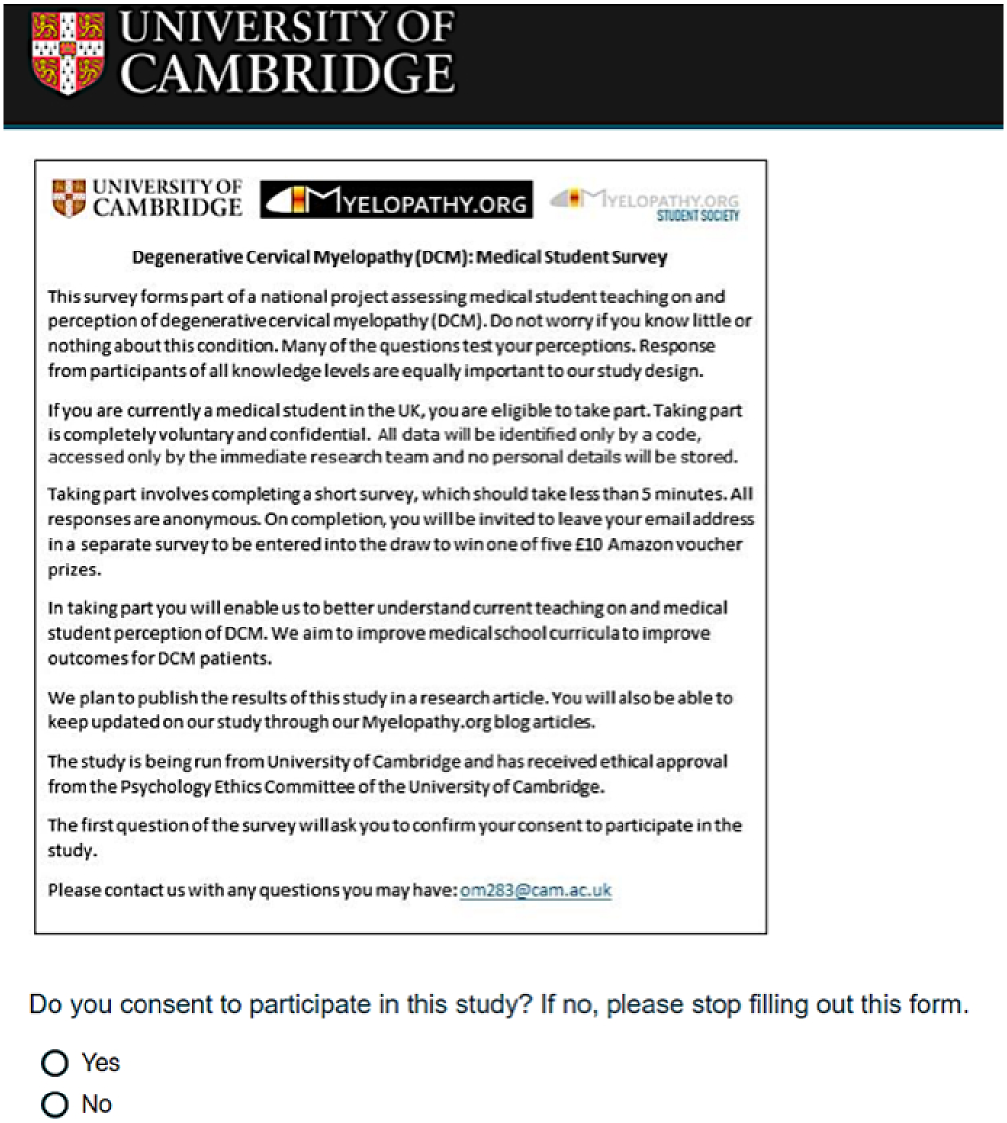

UNIVERSITY OF CAMBRIDGE

UNIVERSITY OF CAMBRIDGE   MYELOPATHY.ORG   MYELOPATHY.ORG STUDENT SOCIETY

Degenerative Cervical Myelopathy (DCM): Medical Student Survey

This survey forms part of a national project assessing medical student teaching on and perception of degenerative cervical myelopathy (DCM). Do not worry if you know little or nothing about this condition. Many of the questions test your perceptions. Response from participants of all knowledge levels are equally important to our study design.

If you are currently a medical student in the UK, you are eligible to take part. Taking part is completely voluntary and confidential. All data will be identified only by a code, accessed only by the immediate research team and no personal details will be stored.

Taking part involves completing a short survey, which should take less than 5 minutes. All responses are anonymous. On completion, you will be invited to leave your email address in a separate survey to be entered into the draw to win one of five £10 Amazon voucher prizes.

In taking part you will enable us to better understand current teaching on and medical student perception of DCM. We aim to improve medical school curricula to improve outcomes for DCM patients.

We plan to publish the results of this study in a research article. You will also be able to keep updated on our study through our Myelopathy.org blog articles.

The study is being run from University of Cambridge and has received ethical approval from the Psychology Ethics Committee of the University of Cambridge.

The first question of the survey will ask you to confirm your consent to participate in the study.

Please contact us with any questions you may have: om283@cam.ac.uk

Do you consent to participate in this study? If no, please stop filling out this form.

- Yes
- No

Figure 5. Survey Front Page including Patient Information Sheet and Capturing of Informed Consent.
Reproduced with permission from Myelopathy.org.
